# A review on machine learning algorithms for the ionic liquid chemical space†

**DOI:** 10.1039/d1sc01000j

**Published:** 2021-05-06

**Authors:** Spyridon Koutsoukos, Frederik Philippi, Francisco Malaret, Tom Welton

**Affiliations:** Department of Chemistry, Molecular Sciences Research Hub, Imperial College London White City Campus London W12 0BZ UK t.welton@imperial.ac.uk; Department of Chemical Engineering, Imperial College London South Kensington Campus London SW7 2AZ UK

## Abstract

There are thousands of papers published every year investigating the properties and possible applications of ionic liquids. Industrial use of these exceptional fluids requires adequate understanding of their physical properties, in order to create the ionic liquid that will optimally suit the application. Computational property prediction arose from the urgent need to minimise the time and cost that would be required to experimentally test different combinations of ions. This review discusses the use of machine learning algorithms as property prediction tools for ionic liquids (either as standalone methods or in conjunction with molecular dynamics simulations), presents common problems of training datasets and proposes ways that could lead to more accurate and efficient models.

## Abstract 2

In this review article, the authors discuss the use of machine learning algorithms as tools for the prediction of physical and chemical properties of ionic liquids.

## Graphical abstract

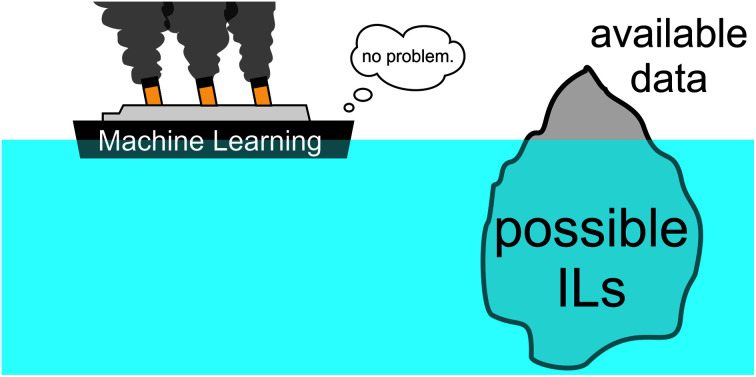

## Introduction

Over the past decades, ionic liquids (ILs) have been a topic of intensive research worldwide. A simple search of the term “ionic liquids” at the Web of Science shows thousands of new papers being published each year, with almost 9000 papers being published in 2020, even excluding the newer trend for Deep Eutectic Solvents. This phenomenon is very much expected, considering that there is a worldwide need to increase the efficiency of industrial processes, while reducing their ecological footprint.^1^ ILs are highly promising materials for this goal, as they can be fine-tuned to fit the needs of a specific application, while their thermal and chemical stabilities and negligible vapour pressures make them easily recyclable. According to numerous studies, ILs can be ideal candidates for a plethora of different applications such as reaction solvents, catalysts, lubricants, electrolytes, extraction media, drug delivery systems *etc.*^2–5^

The synthetic flexibility associated with ILs has led to them being described as ‘designer solvents’.^6^ However, throughout their history there has been insufficient understanding of how the properties of ionic liquids arise from the molecular structures of their constituent ions. Until recently, the usual way of studying and understanding the properties of ILs was essentially by trial and error. Researchers, based on their empirical knowledge and intuitive understanding of ILs and their properties, conceptualised a combination of anions and cations that could have the desired properties and then made homologous series of ILs – hoping that even if the initial attempt was fruitless they would get sufficient feedback to achieve the required properties with a second attempt. However, this method is time-consuming and expensive. Therefore, the need for a prediction, or at least an initial estimation, of the emergent properties of any IL based solely on the structures of its ions becomes evident. Many experts on ILs have indicated that the significant lack of physical data impedes their industrial commercialisation.^7^

Structure–Property Relationship (SPR) has been studied for many years, with major applications being polymer and pharmaceutical research.^8–10^ SPR has been studied from early in IL research, since the natures of the anions and cations, and the interactions between these are usually directly translated to the IL's physical properties.^11^ Therefore, there is a quite extensive qualitative understanding of the basic properties of very popular IL families, which makes it easy for the researchers to find an IL with ‘low melting point’, ‘a wide electrochemical window’ or ‘increased hydrophobicity’. However, in practise the knowledge of general physicochemical characteristics of an IL family is not sufficient when the researcher wants to design tailor-made ILs for specific applications. In this case an accurate prediction of the properties is required that goes beyond the generalities of ‘low viscosity’ or ‘high conductivity’. There is the need for quantitative structure–property relationship (QSPR) studies and the creation of mathematical models that can predict accurate numerical results based solely on structural data of the IL.^12,13^

QSPR for ILs is a difficult and computationally challenging research area, something that can be understood from the fact that there are fewer available predictive models than for other commonly used chemicals (such as pharmaceuticals or molecular solvents). The difficulty lies in the complexity of inter- and intramolecular interactions and that these interactions are not completely understood for all types of ILs. Every experimentalist researcher of ILs has experienced making ILs that don't behave as they expected. This can result in modifying the existing theories in order to rationalise and include those outliers – a process which can prove extremely time consuming – or often to that particular IL being excluded from future studies.

In 1952, computer scientist Arthur Samuel created his famous checkers playing program, introducing a new era for Computer Science, the field of artificial intelligence.^14^ Samuel's checkers player was the first program that could learn while it was running and become a better player after each game. The idea that a program could evolve on its own, without the need of manual modifications on the code, was a technological milestone that would have a major impact in the evolution of Computer Science. Fast forward to the 21^st^ Century, and the evolution of the calculation power of modern computing systems has given machine learning methods (ML) the capacity to perform complicated calculations with extreme time and resources efficiency which are being used by major technological companies.^15^ There are many detailed manuscripts on the history and evolution of ML, some indicative works are cited here.^16,17^

**Fig. 1:**
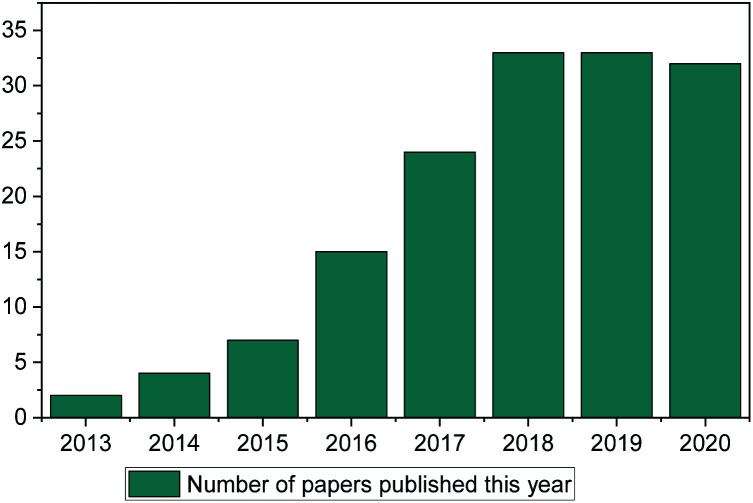
Web of science search of “Ionic Liquids” and “Machine Learning” (search January 2021).

ML methods are currently being implemented in research in a wide range of scientific fields, including chemical discovery and molecular design.^18^ The secret behind their popularity is that in a space of unlimited molecules and synthetic pathways, ML can use complex statistical systems to provide the researcher with a view of greater possibilities to guide their research.^19^ In contrast to other fields (such as drug discovery, toxicology research, synthetic pathways *etc.*) ML has only been used in IL discovery over the past decade, with only a small number of published papers (Fig. 1). This is the main point of discussion of this review paper. Why in an otherwise very much computer-aided research field (there are thousands of available papers on molecular dynamics, Monte-Carlo, *ab initio etc.* calculations) is there so limited literature on ML methods for properties prediction?

## Presentation of the ML methods used in IL research

In order for this work to be helpful, we have to present some short definitions and descriptions of significant terms that will be very frequently used below. Artificial Intelligence (AI) is a term which, nowadays, it is being widely used – without being followed by a strict definition. According to the very popular textbook by Russel and Norvig, AI refers to the “creation of human-like behaviour which can plan, learn, perceive or process a natural language”.^20^ The term intelligence as applied to computers is different to intelligence as it is used in the everyday world. An intelligent machine is not necessarily one that can perform very difficult calculations, but rather a machine that gets feedback from the results it produces and re-uses these in order to continuously improve its methods.^21^

**Fig. 2:**
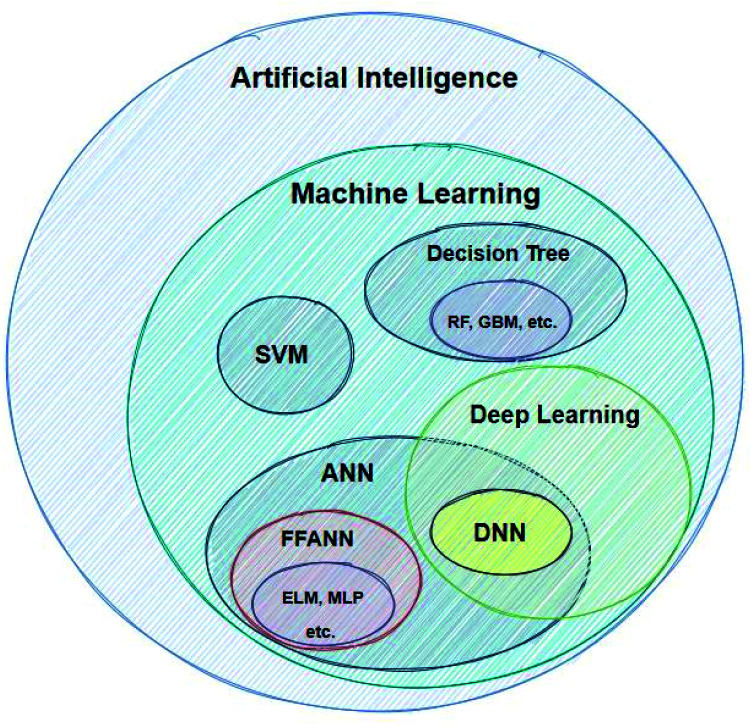
Categorisation of AI computational methods discussed in this work.

Machine learning refers to the creation of algorithms, a sequence of guidelines that help the computer to solve a specific task, sorting and correlating enormous amounts of data. ML offers the computer an automated step-by-step learning capability, enabling it to perform complicated tasks that the user could not program by hand.^22^ These algorithms use statistics in order to correlate large data sets. Input data are fed to the ML learning algorithm, which by using a so-called task-specific feature extractor creates a series of constructed artificial features. The artificial features, which do not necessarily correspond to physical properties of the chemical system being studied, become the input for the regression algorithm (or classifier), which tries to correlate these with the studied property (modelling). There are a great number of different techniques developed for modelling, such as Support Vector Machines (SVMs), Artificial Neural Networks (ANNs) *etc.*^23^Fig. 2 shows the AI methods that are discussed in this work.

**Fig. 3:**
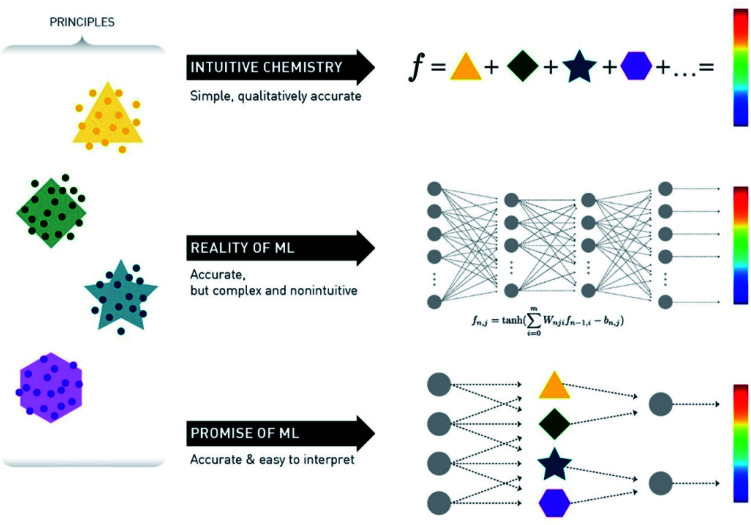
Schematic representation of the promise *versus* reality of the use of ML for chemical reaction prediction. Reprinted with permission from Kammeraad *et al.*^25^ Copyright 2020 American Chemical Society.

A crucial point in ML methods is data representation. For many years the bottleneck of ML research was the construction of feature extractors that could transform raw data to a format suitable for the algorithm. This led to the discovery and flourishing of Deep Learning (DL) techniques, which are methods with multiple levels of representation of data.^24^ Raw data go through multiple non-linear nodes, which transform the initial representation to another – usually more abstract – form, which then makes it much easier for the algorithm to fit very complex equations (Fig. 3).

In order for the reader to better understand the advantages and limitations of the methods discussed further below, we believe it is crucial to have an adequate understanding of the concepts of over- and underfitting. Most regression models are not supposed to go through all the given data points, instead they are creating the curve with the minimum possible residual distance from the measured points.^26^ Overfitting is the modelling error that occurs when the function is fit too closely to a limited set of data and it is a common problem when an algorithm creates an excessively complex model (with too many parameters). As a result, the model picks noise or random fluctuations and considers them as parts of the function. On the other hand, underfitting refers to the case when the created function can't capture the complexity of the data space and wrongly over-simplifies it. An underfit model can neither model the training data nor create/predict new data points.^27^

The obvious question arising from this discussion is “how many parameters are enough?”. This is not an easily-answered question, as this really depends on the complexity of the contributions to the phenomenon being investigated. Enrico Fermi in 1953 was asked whether he was impressed with the agreement between his measured data and computationally calculated values performed by other groups. In his reply he quoted Johnny von Neumann saying ‘with four parameters I can fit an elephant and with five I can make him wiggle his trunk’.^28^ This anecdote has given rise to a debate among theoreticians, trying to prove whether it is actually possible, but has indicated a very significant point of computational research, that the complexity or arbitrariness of parameters can play a crucial role in statistical fitting of measured data.^29,30^

**Fig. 4:**
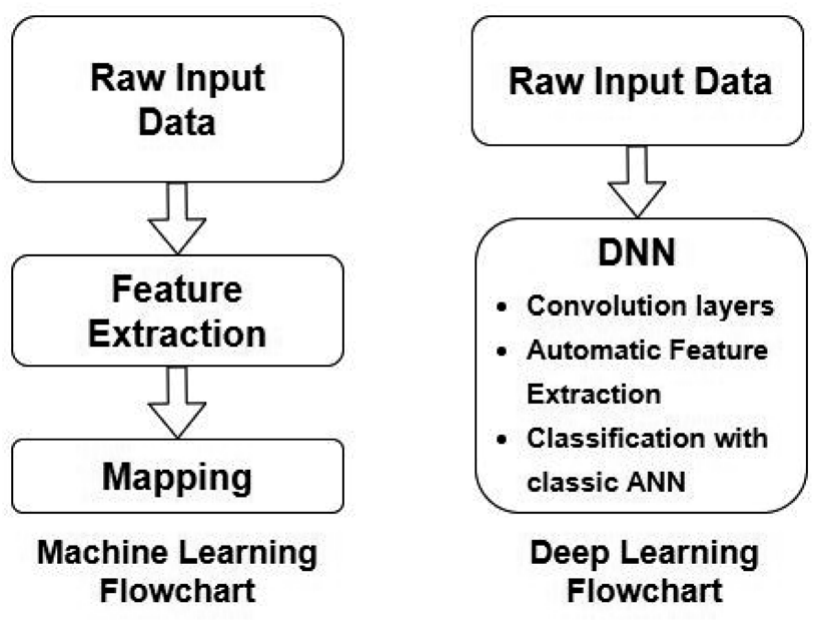
Comparison between conventional ML and DL workflows. Redrawn from Visvikis *et al.*^33^

**Fig. 5:**
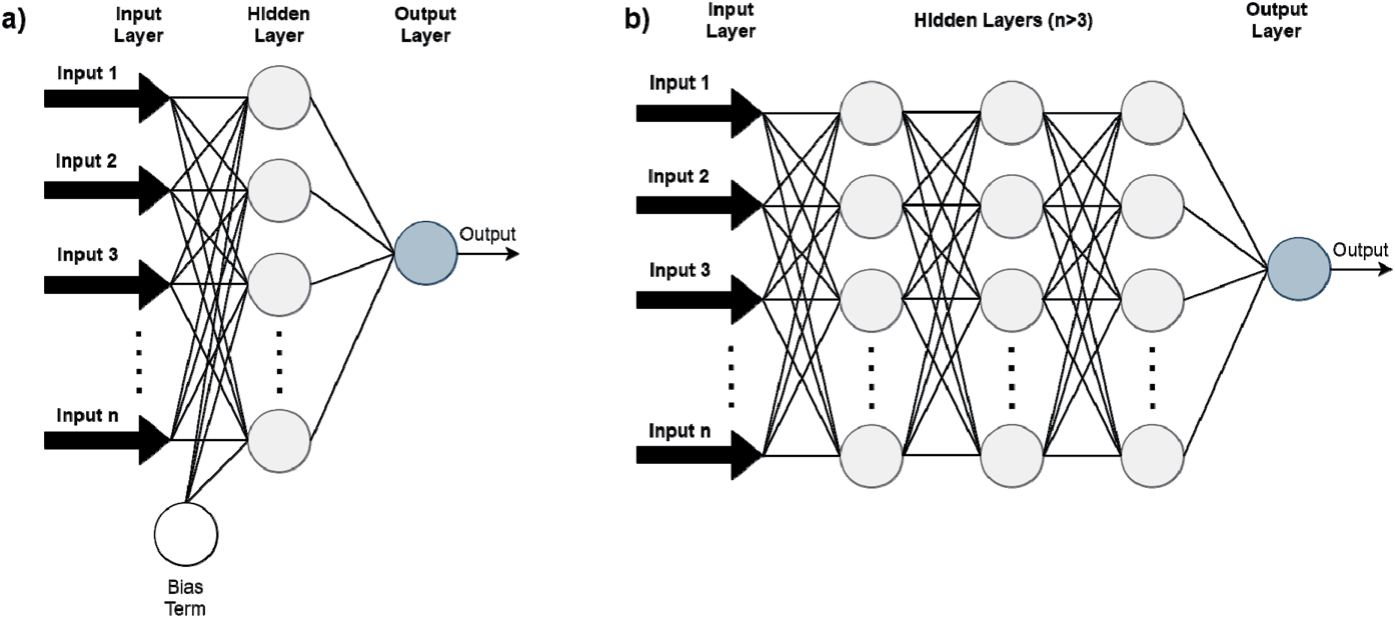
Conventional feedforward ANNs (FFANN) (a) differ from DNNs (b) by having only one hidden neuron layer. Bias terms (output of the NNs when input is zero) are not connected in DNN for simplicity.

Artificial Neural Networks (ANNs), which constitute the basis for most DL algorithms, consist of large successive layers of processing units which lead to different levels of representations and therefore different levels of learned abstraction (see Fig. 4 and 5).^31^ Conventional ANNs get as input the artificial features from the raw data and layer after layer, try to correlate these with the studied property – until they reach the final layer which is property prediction.

Advances in DL algorithms have led to further evolution of ANNs: Deep Neural Networks (DNNs). These methods learn specific patterns extracted directly from the raw data (automatic feature extraction), rather than the extracted features used by conventional ML methods. Furthermore, they are more computationally efficient in finding non-linear correlations. Following the principles of DL, non-linear transformations can be applied from one layer to the next and so on, thus creating an algorithm that can more easily learn more abstract features.^32^ Although they are not identical, the terms ANN and DNN are often interchanged in the literature, making it difficult for a reader with limited knowledge of the subject to directly understand the used method.

However, DNNs have their flaws, which have to do mainly with the existence of many hyperparameters, parameters whose values define the network's structure and guide the training process, which require a lot of computational time and effort to fine-tune. Moreover, because of the numerous layers and their incredible correlation capacity, they are very vulnerable to overfitting the data – as they tend to recognise and model rare correlations that appear in the dataset, but might not actually have physical significance.^34^

Although ANNs are arguably the most widely used AI technique in chemical research (and many other fields), they do have their flaws and some researchers look for alternatives. The most significant disadvantages relevant to chemical research are the strong dependence between input and output, long training times with many epochs (number of passes of the training set completed by the algorithm), the need for very large and diverse datasets and their susceptibility to overfitting.^35,36^ Trying to overcome these problems, many researchers turn to Support Vector Machines (SVM), which at least in the case of IL property prediction, is the second most popular method of choice.

SVMs work on the simple rule of depicting the training data as vectors in space and trying to categorise these with the widest possible gap between them. New (unseen) data are plotted in the space and they are integrated into either of the categories based on the side of the gap to which they belong.^37^ SVMs investigate the possible hyperplanes, a space of N-dimensions which offers maximum separation between two categories, through various non-linear transformations, in which the given data will be linearly separable and then translate this separation to the initial training space.^38^ These models are able, in short times and with smaller datasets than ANNs, to solve problems related with data classification and regression. However, they require the solution of quadratic equations in order to effectively describe a given dataset. Simplification of the problem comes by transformation of the quadratic equations to linear using the least-square method (LSSVM), thus reducing the system to a set of 2*N* + 2 equations with 2*N* + 2 variables (*N* is the number of provided data points).^39,40^ The LSSVM approach has turned SVMs from classification to regression algorithms capable of reportedly very high precision and higher possibilities of reaching a global minimum, in comparison to ANNs which very often terminate at local minima of the equations.^41,42^

**Fig. 6:**
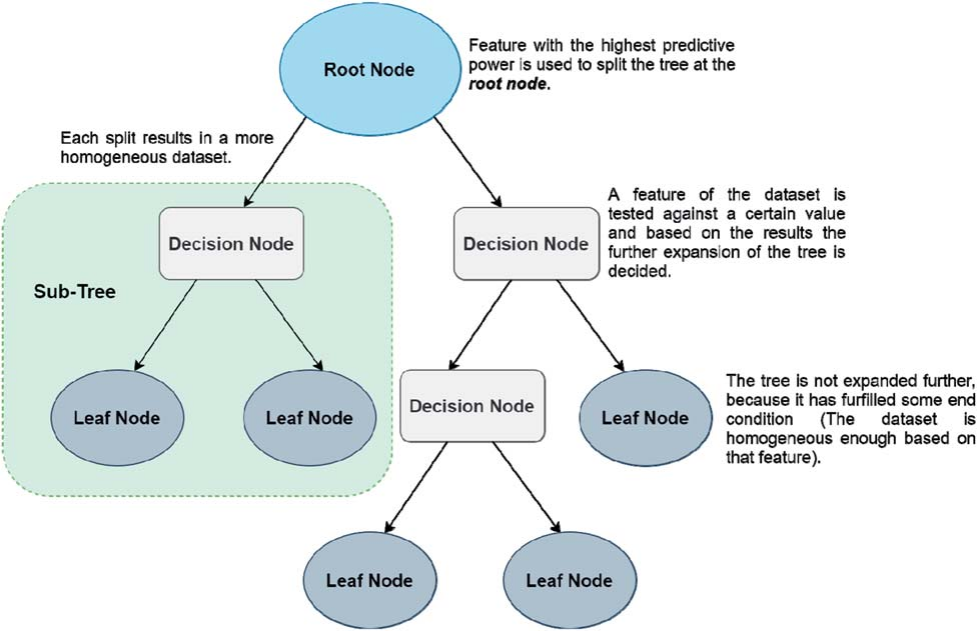
Structure of a simple DT.

The last ML method that will be discussed in this work are Decision Trees (DTs). DTs have gained popularity because of their simplicity and efficiency in dealing with high dimensional data, but they are weaker in prediction accuracy than the methods described above. There is a plethora of QSPR studies using classification and regression decision trees (CARTs), usually on datasets with many different molecular descriptors.^43–45^ The creation of a CART is based on a very simple method. Initially a tree is created by partitioning the initial data points (root node) to ‘child’ (or leaf) nodes. The aim of this step is for every created child subgroup to be more homogeneous than the ‘parent’. DTs are very prone to overfitting, therefore it is quite usual for researchers to create trees with a very large number of nodes, in order to avoid that problem. However, this results in many nodes being ‘weaker’, *i.e.*, not useful to the system. Then comes the second step, which is pruning, with the aim to remove any unnecessary splits of the tree. Unlike NNs, DTs don't use artificial features, but the predictive features correspond to actual chemical parameters (such as HOMO/LUMO energies, molecular volumes, molecular weight *etc.*) Finally, the CART with the lowest error on a test set prediction is selected as the optimal tree. Prediction of a property reaching a terminal leaf node is calculated as the average value from all training set points that have reached the same node.^46,47^ A simple form of a DT algorithm is shown in Fig. 6.

DTs have a fundamental disadvantage in that a simple tree structure suffers from a large bias, while a complex tree has a large variance. In order to overcome these problems, researchers use various ensemble methods, which try to group together many simple trees (week learners) in order to create a strong learner.^48^ There are two basic categories of ensemble methods, bagging and boosting. Bagging aims to reduce the variance of a DT by splitting the training data to subsets, training different trees and use an average of those models – which has proven as more efficient than a single DT. Random Forest (RF) is an extension of bagging, which as an addition uses a subset of the existing predictive features, instead of using all of them to grow the trees. RF offers the advantage of handling better high dimensional data.^49^ Boosting is the ensemble method which creates a sequence of many simple trees (weak learners) in order to achieve one strong learner. Each tree is focused on reducing the fitting error received from the previous tree. Gradient boosting is an extension of this method, which uses of a loss function that detects residuals. New learners fit to the residuals from previous steps, trying to recover the loss (difference between actual and predicted value) so that the model improves faster.^50^ A basic advantage of the Gradient Boosting method is that it supports the use of different kinds of loss functions (higher versatility) and also it provides accurate results even if there are interactions between the studied parameters.^48^

## ILs as input data

When QSPR models are being set up, a major decision point is how the researchers will translate chemistry to maths. QSPR correlations can use input data either directly from experimental measurements or create descriptors based upon the molecular structure. The vast majority of properties prediction methods (both classical computational and ML methods) for complex molecules are based on group-contribution theory (GC).^51–54^ GC models break down molecules into characteristic sub-structures (descriptors), which then can be correlated with specific effects on the compound's properties. The simplest, and most used, GC models study first-order correlations between the model and the studied property, in which the property arises as a simple sum of the contributing factors. Over recent years, the increase in the available computational power has made more complicated second and third order models more popular (eqn (1)).^55^

1 *f*(*X*) = ∑*N*_i_*A*_i_ + ∑*M*_j_*B*_j_ + ∑*W*_k_*C*_k_

*f*(*X*) is the value of a studied property *X* at given conditions (*e.g.* viscosity at given temperature and pressure), *A*_i_, *B*_j_ and *C*_k_ are first, second and third order contribution factors – corresponding to the number of performed regressions, *N*, *M* and *W* show how many of each factor appears in a molecule.

The number of descriptors and the complexity of chemical structures are significant parameters that affect the results of the model, but they are decided on a trial-and-error basis with each researcher following a different route. Especially for IL systems, the occurring interactions are numerous and complex. Therefore, there are limitations on how accurately somebody can depict an IL with such descriptors.^56^ ML models are usually data-hungry and if not properly adjusted they tend to create hundreds of parameters and overfit the results – something that should be avoided by all means. The general rule is that the model should remain as simple as possible, to give meaningful predictions and as general as possible, in order to be able to encompass a large range of molecules. Moreover, there are studies that show that, in some cases, increasing the order of the correlation factor makes the models more complicated but does not actually improve their accuracy.^57^

Another method of transforming chemical structures to descriptors was introduced by Valderrama *et al.*^58,59^ Their mass connectivity index (MCI) offers the capability, by using simple calculations, to connect the mass of the functional groups in a molecule with the type of connection (branching, double bonds *etc.*). However, the simplicity of the method comes with the limitation of not being able to define intermolecular interactions (such as hydrogen bonding) to the index, which is important for finding QSPRs in ILs. They used their MCI as an input descriptor for a neural network that predicts viscosity with promising results for a small range of studied ILs.^60^ However, apart from their works, MCI has not been used as input for any of the other ML studies for ILs.

Molecular descriptors, such as those discussed above, present the limitation of requiring researchers to find sets of relevant descriptors for each case and also usually they have to deal with high dimensional data. In order to overcome those problems another category of methods has been created, which works directly on molecular structures. Graph-convolution NNs transform the molecular structures to a set of neural fingerprints, which are used in order to translate structures to graphs (vectors).^61^ A popular representation of structures uses graph nodes to represent atoms, while the edges describe bonds.^62^ Graph theoretical approaches have been used to describe and analyse various different chemical systems.^63–65^ The used network can be set in order to optimise the efficiency of extracted characteristics, thus improving the accuracy of the model. There are few published works on graph-based frameworks for encoding chemical structures for ILs, however these works tend to focus solely on one family of anions or cations and therefore their extension and generalisation might still be limited.^66–68^

Another family of descriptors used in QSPR methods are those of quantum chemical (QC) or thermodynamic nature. QC descriptors use values from quantum calculations, such as HOMO and LUMO energies, polarity, electron affinity, electronegativity *etc.*^69–71^ Similar to the other techniques, a variety of such descriptors are calculated for a dataset of ILs with known properties and then correlation methods are used to choose those which appear to have more significant relations to the properties.^72^ Based on QC descriptors theory, some studies have used descriptors based on COSMO-RS σ-profiles (molecular surface charge distributions).^73^ COSMO-RS offers the capability of property estimation, which however requires DFT calculations that usually run on high performance computing systems.^74,75^ Unlike DFT calculations, a pre-trained ML algorithm might be able to run on an average office computer. Stocker *et. al.* recently published a very interesting study about the use of ML in chemical reaction networks, which shows that the prediction of new data points using ML methods is performed much faster than with DFT calculations, with equal accuracy.^76^ Using COSMO-RS σ-profiles as data for ML methods, seems promising and has been implemented in various classical property regression models with very promising results,^77–79^ but so far with only few implementations to ML algorithms.^80–85^

## Prediction of physical and chemical properties of ILs

As discussed above, ML methods are superior *versus* classical data analysis techniques in two main aspects, data classification and prediction. The predictive ability of these algorithms is being investigated in depth in ILs research, with the main aim being the accurate prediction of physical and chemical properties. Viscosity, density, melting point, toxicity and solubility of harmful gases are properties of that have been of particular interest, as they are process-relevant and can lead to the design of new commercially usable ILs with respect to the demands of Green Chemistry and Sustainability. Below we present the existing studies on ML for the prediction of various properties in ILs; details about the families of the studied of ILs, as well as the number of training and test datasets can be found in Tables 1 and 2.

**Table 1:** Summary of works using ML methods for prediction of properties in IL

Property	IL family	Method	Distinct ILs	Training/test set points	Ref.
Viscosity	Im, Py, Quin, Pyr, Ox, Pip, Mo, Azp, Guan, N, P, S, dicationic	FFANN	1484	11031/613	53
	Im, Py, Pyr, N, P	FFANN	81	654/81	96
	Im, AA, N, Guan, Quin, Mo, Ox, P, Pip, Py, Pyr, Pyrr, S	LSSVM	443	1254/418	40
	Im, Py, Pyr, P, Quin, N	FFANN	66	612/124	99
	Im, Py, Pyr, P, N, Mo, Pip, S	ELM (FFANN)	89	1205/297	100
	Im, Py, Pyr, P, N	MLP (FFANN)	33	651/72	163
	Im, N, Py, Pyr, P, Pip, Mo, S, Cprop, Azp, Guan, Trz, Bic, Pz, Thur, Quin, thz, amd, ox, pipz, tetraz	FFANN and LSSVM	1974	1437/159 and 4479/453	97
	Im, Py, N	FFANN	31	327/31	60
Density	Im	MLP (FFANN) and RBF	n/a	317/68	93
	Im, N, Py, Pyr, P, Pip, Mo, S, Cprop, Azp, Guan, Trz, Bic, Pz, Thur, Quin, thz, amd, ox, pipz, tetraz	MLR, FFANN and LSSVM	1999	5632/625	94
	Im, Py, Pyr	FFANN	50	399/83	54
Melting point	Trz, Pyr, Py, Pip, P, Mo, Im, N, S	PLSR, SVM, RF, GBM and k-nn	2212	1486/726	88
	Im, Py, Pip, P, N	FFANN	62	50/12	87
	Im	Regression trees and SVR	281 and 134	225/22 and 107/13	90
	Trz, Pyr, Py, Pip, P, Mo, Im, N, S	KKR	2212	1770/442	92
	Im, N, P, Py, Pyr, S	PLSR, GBM, Cubist, RF, CART	467	1646/1501	164
	Py	FFANN, DT	126	n/a	47
	Guan	CPG NN	101	81/20	86
	Py	RNN	126	84/42	67
Surface tension	Im, Py, P	FFANN	79	616/132	165
Toxicity	Im, Py, Pyr, P, N, Pip, Mo, Quin, S	GFA and LSSVM	270	203/67	116
	Im, Py, Pyr, Pip, N, Quin	ELM (FFANN)	119	100/19	118
	Im, Py, Pyr, Pip, P, N, Quin	MLR and ELM	160	128/32	120
	Im, Py, Pyr, Pip, N, P, Mo	CCN and SVM	292	204/88	115
	Im, Py, Pyr, Pip, P, N, Mo	ELM	142	113/29	121
CO_2_ solubility	Im, N, P	MLFNN (FFANN)		144 (pre-trained on H_2_S)	102
	Im, P, Pyr	MLP and ANFIS	14	546/182	101
	Im, N, Py, Pyr	MLR and LSSVM	21	16/5	103
	Im, N, Guan, Py, Pyr, P, Ur	PLSR, CTREE and RF	158	5424/5424	71
	Im, P	LSSVM	11	128/385	104
	Im, P	MLP	20	907/208	105
	Im, Pyr, P	DNN, RNN and CNN	13	n/a (ratio 7/3)	106
	Im, Pyr, P	MLP	13	595/149	166
	Im, PY, Pyr, P, N	LSSVM, MLR, RF and DT	36	1241/414	108
	Im, Py, Pyr, Pip, N, P, S	FFANN and SVM	124	8093/2023	107
H_2_S solubility	Im, N, P	MLFNN (FFANN)		513/165	102
	Im	MLFNN (FFANN)	11	372/93	109
	Im, N	ELM (FFANN)	37, 27	1025/257	84,110
	Im	ANFIS, MLP, RBF	13	554/1140	111
	Im	LSSVM	9	590/62	112
	Im	SGB (DT)	11	369/96	113
	Im, N	ELM (FFANN)	28	1055/263	114

### Physical properties

#### Melting point

Carrera *et. al*.^47^ (2005) predicted the melting points of pyridinium bromide salts, using DTs and a NN. The structure of each IL was represented in the DTs using a sum of 1085 molecular descriptors. The descriptors chosen by the trees as more significant were investigated for their ability to train a NN. The prediction results were not very accurate (deviations of around 40 °C between tested experimental and predicted melting points), but this was one of the earliest works that showed that ML methods can be very promising for the prediction of IL properties. Following up on their research, in 2008 they published another work^86^ predicting the melting points of guanidinium ILs. This work included a NN with a similar structure as the one studied before. The structures of ILs were represented by a set of 184 molecular descriptors. It is important to recognize here that this is one of the very few works, in which the team synthesized a set of new ILs to test the accuracy of their results. The comparison showed differences up to 70 °C between the predicted and experimental melting points.

Bini *et. al*.^67^ (2008) worked on the same set of pyridinium bromide salts as the earlier work by Carrera *et. al*., using a recursive neural network (RNN). In this work, the researchers used graph convolution theory to avoid the manual creation of input descriptors. The accuracy of prediction was similar to that of Carrera *et. al*., but their work significantly reduced the required effort to translate the molecular structure to format understood by a computer.

Fatemi and Izadian^87^ (2012) used a multilayer perceptron NN (MLP-NN), which is type of ANN that is trained more easily on nonlinear correlations. A set of 62 ILs from various families (see Table 1) was investigated, using molecular descriptors as input data for the NN. The study showed improved accuracies compared to earlier studies, but the authors state that MLP-NNs are useful only in the cases that accuracy is preferred over speed.

Venkatraman *et. al*.^88^ (2018) investigated both linear and nonlinear approaches for the prediction of the melting points of different families of ILs, using DTs and SVM models. They used a bespoke training set of more than 2000 ILs extracted from selected papers, which they transformed to computer input using quantum mechanical descriptors obtained by computationally low-cost PM6 calculations. They compared their results to the prediction model provided by COSMO-RS. This study showed moderate absolute accuracy, but behaved well when predicting relative differences or trends in melting point differences. Following up on their study, in 2019 the same group published an extensive library of property prediction (including, but not limited to, melting point, viscosity, glass transition temperatures, density *etc.*).^89^ The prediction was based on variety of different ML methods, from which the best performing model on each property was selected. This work is very important for property prediction, as they have created a pool of over 8 million ILs predicted properties, which can be used for guided synthesis of task-specific ILs (always taking into account possible accuracy limitations).‡‡We tested the deviation of the published library's prediction to experimental results on two ILs recently published from our group,^284^ namely [N5551][NTf_2_] and [P5551][NTf_2_] and the comparison of the experimental and predicted values (given in parentheses) are for [N5551][NTf_2_]: density at 25 °C: 1156 (1206 ± 14) kg m^−3^, viscosity at 25 °C: 480 (452 ± 136) mPa s, melting point: 37 (18 ± 29) °C, and for [P5551][NTf_2_]: density at 25 °C: 1152 (1246 ± 14) kg m^−3^, viscosity at 25 °C: 206 (264 ± 60) mPa s, melting point: 20 (62 ± 50) °C.

Cerecedo-Cordoba *et. al*.^90^ (2019) noticed that the published works until then on ML for IL property prediction were not significantly more accurate than classic QSPR methods and hypothesized that this was due to the struggle to deal with many different types of IL families at once. Therefore, they decided to work solely on imidazolium ILs. They created a framework based on different clustering methods and simple regression models, from which in each case the best combination was selected. The authors claim that the clustering architecture can predict the melting point of those ILs better than other proposed models and offers the advantage that this can be easily expanded to any IL dataset and property. Following up on their research, in 2020 they created NeuroFramework,^91^ a framework that trains NNs that can be used for the prediction of the melting points of ILs. Similar to their previous research, this framework, although tested for melting points, can be expanded to any property.

Low *et. al*.^92^ (2020) investigated the effect of descriptor choice on melting point prediction. They used Venkatraman's dataset of 2200 ILs and quantum mechanical descriptors. After trying different combinations of models and descriptors they concluded that the most accurate model shows a deviation between predicted and experimental melting points of around 30 °C, and the absence of structure-related descriptors means that given a suitable training set, the model can be used for any family of ILs.

#### Density

The work of Valderrama *et. al*.^54^ (2009) is one of the earliest works for the prediction of IL densities using ML that combines GC theory with an ANN. This study considered only 25 possible functional groups, therefore the number of possible cations and anions for study was quite limited. The accuracy of this method was very good for test data excluded from the training set, while for other – completely unknown structures – acceptable accuracy for engineering calculations can be achieved.

Najafi-Marghmaleki *et. al*.^93^ (2016) used two different ANNs to predict the densities of neat ILs and IL-water mixtures, for various imidazolium ILs. The authors compared their two methods, which have very similar prediction accuracies. This work presented a slightly different scope compared to the other published works, the need to model not only the properties of pure ILs, but also of their mixtures – since they are used very often in chemical research.

Paduszyński^94^ (2019) created a database of predicted IL densities based on a combination of three different ML methods, MLP, FFANN and LSSVM. The author claims superior accuracy compared to the, by then, state-of-the-art model.^95^ It is worth noting that both the training dataset (more than 2000 ILs) and the methodology followed by Paduszyński are more complex than those presented previously, both of which contribute to the improved results.

#### Viscosity

Valderrama *et. al*.^60^ (2011) were one of the first groups to investigate the prediction of viscosity trends in ILs. They trained an ANN using their MCI as input (see subsection ILs as input data) and testing the result in 26 ILs – mostly based on the imidazolium cation family. The results were satisfactory, leading to general deviations less than 5% of the experimental value and showing that MCI can indeed be used as input parameters for IL property prediction.

Dutt *et. al.*^96^ (2013) followed another path, although also using an ANN; they didn't include any structural features as input data, instead they only provided as input the logarithm of viscosity at 323.15 K and the inverse of the reduced reference temperature. They compared their results to commonly used empirical viscosity equations (such as Vogel–Tamann–Fulcher and linear Arrhenius model) and concluded that the ANN offers the advantages of showing lesser overall residual errors and that they don't seem problematic in any specific ion family.

Paduszyński and Domańska^53^ (2014) were the first to combine GC theory with ANNs for the prediction of viscosities of ILs, based on a database of 1484 ILs. Their study showed that the worst accuracy was obtained for ammonium and dicationic ILs, which they attributed to the lack of sufficient data for these IL families. In 2019 Paduszyński^97^ extended his model, to include data from more than 2000 ILs, using a combination of FFAAN and LSSVM models. This method showed superior prediction capacity compared to classical QSPR methods, but the author recognised that interpretation of the parameters as understandable molecular properties is unfeasible. In 2021 the methodology was extended to the prediction of surface tension of ILs.^98^

Fatehi *et. al*.^99^ (2017) noticed that many of the so far proposed methods required other experimental measurements as input data (such as density) and thus they created an ANN which aimed to predict viscosities of pure ILs based solely on their molecular structures. Moreover, unlike the methods presented above, their algorithm considered the effect of pressure on the ILs' viscosities. The algorithm showed a good fitting to both training and test data for the studied IL systems, with authors claiming that it can be expanded to other similar systems.

Kang *et. al.*^100^ (2017) used a newly-discovered extreme learning machine (ELM) algorithm to predict viscosities of ILs, using σ-profile descriptors as input data. ELM is a FFANN algorithm which benefits from fast learning speed and good generalisation capabilities. The study showed very interesting results and proved that the viscosities can be adequately predicted in a wide temperature and pressure range with no structural input data, using only thermodynamic data.

Baghban *et. al.*^40^ (2017) used a LSSVM model, implementing GC theory. This model represented the structures as a sum of 46 pre-determined substructures in the molecule and required temperature as an input. Unlike Fatehi *et. al.* this model doesn't take into account the effect of pressure on the viscosity of ILs, but follows the same general idea of predicting the property based solely on structural data.

### Solubility of gases

#### CO_2_ solubility

Baghban *et. al*.^101^ (2015) investigated the CO_2_ solubility in a selection of 14 ILs using an MLP-ANN and compared the results with those obtained from classic thermodynamic equations, such as Peng–Robinson and Soave–Redlich–Kwong. Thermodynamic properties of the ILs (such as critical temperature and pressure§§For CO_2_ and H_2_S solubility studies, many researchers use experimentally inaccessible critical properties, boiling points or acentric factors of ILs. These properties are in fact calculated from modified Lydersen–Joback–Reid group contribution methods.^285,286^) were used as the model input, without any molecular structure descriptors. The ANN model shows improved prediction accuracy compared to the thermodynamic models, as it uses more complex nonlinear correlations.

Hamzehie *et. al*.^102^ (2015) trained a FFANN to predict the solubility of both H_2_S and CO_2_ in commonly used ILs and amine mixtures. Similar to the previous case, no structural characteristics are provided as inputs for the model, instead thermodynamic properties and the apparent molecular weight of the solution were used. The authors trained and tested their algorithm on H_2_S data and then used the CO_2_ solubility data to test the extrapolation capacities of their method. The results showed that the algorithm has adequate extrapolation capacities that can include different types of gases.

Mehraein and Riahi^103^ (2017) compared the prediction abilities of a multiple linear regression and a nonlinear LSSVM model on CO_2_ solubilities for 21 commonly used ILs. Unlike the methods discussed above, the authors here used molecular structure descriptors as input for their models, after optimising their geometries based on PM6 level of theory. The LSSVM model showed improved results compared to the linear model. This study also provided some useful insight on the structural parameters that affect CO_2_ solubility (based on the significance of the input descriptors), revealing that the cation size, structural asymmetry and the polarity of ions significantly affect the results.

Venkatraman and Alsberg^71^ (2017), similar to their studies on melting point discussed above, used descriptors based on COSMO-RS, in combination with different ML methods, to find the model which can better predict CO_2_ solubility. A RF nonlinear trees ensemble showed improved results compared to other methods, with the predictions however not being equally reliable for all IL families (phosphonium and ammonium ILs showed larger deviations). The authors state that hydrogen bonding and interactions between CO_2_ and ILs should be considered, as they would improve the model, but they are more computationally demanding.

Ghazani *et. al*.^104^ (2018) worked on the prediction of the absorption of CO_2_ containing common gaseous impurities (mainly focused on greenhouse gases). A LSSVM algorithm was trained on experimental data of ternary mixtures containing two gases and an IL, providing as input no structural details for the ILs. The results were compared to other ML methods (RBF-ANN and MLP-ANN) and showed superior performance.

Mesbah *et. al*.^105^ (2018) focused on the prediction of the solubility of CO_2_ and supercritical CO_2_ in 20 common ILs using an MLP-ANN. The authors studied a wide temperature and pressure range, 278–450 K and 0.25–100 MPa respectively. No molecular structure descriptors we used in this model either, the solubility of CO_2_ was expressed as function of molecular weight, critical temperature and pressure of the ILs. The model showed accurate fitting and prediction capacity in a very wide temperature and pressure range, reaching to supercritical CO_2_. The authors note the advantage of their method of achieving high accuracy without the need for any physical data as input.

Deng *et. al*.^106^ (2019) predicted the solubility of CO_2_ in ILs using deep learning methods. They trained three different NNs on CO_2_ solubility data in ILs, using only IL molecular weight and critical properties as input and compared their results to classic thermodynamic models. As expected, the deep learning methods showed improved prediction capabilities than the classic thermodynamic models, showing smaller prediction bias. The authors correctly state that the extrapolation of this model would require larger and more diverse datasets.

Song *et. al*.^107^ (2020) combined group contribution theory with two ML models, an ANN and a SVM. Both models were trained on a large database of more than 10 000 CO_2_ solubility points under different experimental conditions (both temperatures and pressures considered) for 124 ILs. 51 molecular structure descriptors were used in total, with 13 cation cores, 28 anions and 10 different substituent groups. Both ML models showed high accuracies, with the ANN showing slightly better results. However, the authors here note a significant restriction of all ML models, since they are not defined by thermodynamic principles, there is no theoretical guarantee that the produced prediction is not an outlier. The results are purely statistical, which means that there is always a possibility (however low or high this might be) that for a random structure the model will fail.

Aghaie and Zendehboudi^108^ (2020) performed a comparative study between different ML methods and input parameters, in order to identify the optimum model for the prediction of CO_2_ solubility in ILs. The studied models were LSSVM, FT, RF and multilinear regression, each trained and tested on two different datasets, one with thermodynamic data and the second with structural descriptors as inputs. In both datasets RF and DTs show improved prediction capacity compared to the other methods. At the same time, the models with molecular structure inputs were more reliable than those with thermodynamic properties inputs.

#### H_2_S solubility

Shafiei *et. al*.^109^ (2014) used ANNs in order to predict the solubility of H_2_S in 11 common ILs. Only the critical properties of ILs were used as input data for the model. The ANNs were trained on a dataset of experimental measurements, using different training techniques (namely back propagation – BP and particle swarm optimisation – PSO). The PSO-ANN showed better fitting and prediction capacity than the BP method and creates a viable alternative to classic thermodynamic prediction models, as the relative deviations are very similar.

Zhao *et. al*.^84,110^ (2016) used an ELM algorithm, which they trained on COSMO-RS σ-profiles and simple molecular structural fragment descriptors respectively. The authors created an extensive dataset with almost 1300 data points for H_2_S solubility in 37 ILs. Both models showed satisfactory accuracy, with the σ-profile descriptors having the advantage of providing more molecular interaction information, while the molecular fragment descriptors presented an easier alternative for less experienced user.

Amedi *et. al*.^111^ (2016) evolved Baghban's^101^ method for CO_2_ solubility, in order to study the case of H_2_S. In their study they included both binary mixtures of H_2_S + ILs and ternary mixtures of H_2_S + CO_2_ + ILs. The input data and methodology followed was identical to their previously published work, with the MLP-ANN showing again better results compared to the other models.

Fattahi *et. al*.^112^ (2017) used an LSSVM model to predict H_2_S solubility in ILs and mixtures of amines with molecular solvents. The model input variables in this case are temperature, pressure, the apparent molecular weight of the system and the mass concentration of the solutions. Their study showed that molecular weight was the most significant factor of the model. The overall accuracy of the algorithm was adequate.

Soleimani *et. al*.^113^ (2017) used a gradient boosting DT to calculate the solubility of H_2_S in 11 ILs as a function of the ILs' critical properties. The DT's performance was compared to an LSSVM and showed more accurate prediction results. It is known that DTs are advantageous due to the simplicity of their structure, compared to other ML methods, but due to the small range of training and test data no other conclusions can be extracted from this study.

Kang *et. al*.^114^ (2018) used their ELM algorithm, previously tested on IL viscosity prediction^100^ and expanded it to H_2_S solubility. The method was trained on 1300 data points in 28 distinct ILs of various anions and cations. Unlike their previous study, where they used molecular structure descriptors, here they presented new descriptors based on electrostatic potential surface. The advantage of this method, also in comparison to the critical properties required by previously discussed methods, is that no experimental data are needed as input, all the descriptors needed can be theoretically calculated. The model showed high prediction accuracy and presented a viable alternative for researchers who want to get a solubility estimate without running preliminary experiments or physical measurements on the studied ILs.

**Table 2:** Explanation of cations abbreviations presented in Table 1. Structures given in the ESI (see ESI)

Cation names	Cation names
**Im**: Imidazolium	**Cprop**: Cyclopropanium
**Py**: Pyridinium	**Guan**: Guanidinium
**Quin**: Quinolinium	**Trz**: Triazolium
**Pyr**: Pyrrolidinium	**Bic**: Bicyclic
**Pyrr**: Pyrroline	**Pz**: Pyrazolium
**S**: Sulfonium	**Thur**: Thiouronium
**Ox**: Oxazilidinium	**Cs**: Cyclic sulfonium
**Pip**: Piperidinium	**Thz**: Thiazolium
**Mo**: Morpholinium	**Amd**: Amidium
**Azp**: Azepanium	**Pipz**: Piperazinium
**N**: Ammonium	**Tetraz**: Tetrazolium
**P**: Phosphonium	**Ur**: Uronium
**Guan**: Guanidinium	

### Toxicity

Basant *et. al*.^115^ (2015) investigated the acetyl cholisterenase enzyme (AChE) inhibition potential of ILs using SVMs. The input data were coded using Moses Descriptor Community Edition, by choosing 211 molecular descriptors. Out of those descriptors, the ones that had low variance were disregarded. The SVM outputs were compared to previously developed QSPR models and showed higher statistical confidence. Their work helped to identify which structural characteristics of the ILs are mostly responsible for AChE inhibition and also, their algorithm can be trained and generalised for more IL families easily.

Ma *et. al*. published two works in 2015^116,117^ predicting the cytotoxicity of ILs to Leukemia Rat Cell Line (IPC-81) and the ecotoxicity of ILs on *Vibrio fischeri*. The anion and cation molecular descriptors used in their studies were produced by Dragon software and included 0D–3D structural features. In both cases, the results obtained by a LSSVM nonlinear model appeared superior to the linear model, which verifies once more that the structure–property relationship is complex in the IL chemical space and simpler linear models sometimes fail to accurately predict the studied property.

Cao *et. al*.^118^ (2018) used the same dataset to predict the cytotoxicity towards Leukemia Rat Cell Line (IPC-81) using quantum chemical descriptors. They compared multiple linear regression, ELM and SVM algorithms trained on the same dataset. Their study showed that ELM has superior fitting and prediction capacity compared to their SVM (linear regression performed significantly worse than the other two) and also highlighted that the lipophilicity of the cation plays a major role in the cytotoxicity of the IL, although this was known already previously from conventional studies.^119^ Although their results were not significantly improved compared to Ma *et. al*., this study does show that quantum chemical σ-profiles, can be used to model the cytotoxicity behaviour of ILs. Zhu *et. al*.^120^ (2019) expanded this work to AChE inhibition and showed that their ELM methodology can provide accurate results for ecotoxicity of ILs too. In 2020 Kang *et. al*.^121^ further progressed their work on *Vibrio fischeri* by using electrostatic potential surface area descriptors as input, thus improving the accuracy of their previously published algorithm.

## Common issues with datasets

ML correlation methods are highly dependent on the quality of the datasets, this is probably the most significant part of the algorithm, the part that makes training possible.^122^ AI is doomed to fail if the training data are not ‘good enough’. Hence, we discuss below the parameters that make a dataset ‘good’ and how these apply to IL research. As we shall see, the composition of the ILs' literature, which has come about through historical circumstances and was never designed for the purpose of supporting ML approaches, imposes limitations on the generalizability of results.

### Size

Unfortunately, nobody can answer the question “how much data is enough to train a ML algorithm?”, as it significantly depends on various factors, such as the complexity of the model (*e.g.* number of inputs/outputs, the relationship between parameters, the quality of the data). Every algorithm is different and shows different sensitivity to the size of training set. A general practice followed by researchers is to try to get comparable prediction accuracy between the training and the test set. ML algorithms tend to overfit when they lack enough data, but this is not only related to the absolute number of the training data, but also to the diversity of the set, which will be further discussed below.

There are studies on the effect of training set size on QSPR models that show there is no simple correlation between the size of the set and the predictive ability of the model, but it is rather dependent on the studied property.^123^ Obviously, if the training set includes a large percentage (*e.g.* 70%) of the total dataset, then the models usually show high predictive capabilities, but the effect of training set size reduction is not straightforward. Also, as noted by Hughes *et al.*, some properties such as melting points are more difficult to predict than others, in this case because the input descriptors can't properly describe the change in chemical interactions between solid and liquid phase.^124^ For example, it is quite common for ILs that increasing the alkyl chain length has complex effects on the melting point, with even the direction of effect being different for shorter or longer chains, due to different preferable interactions or molecular arrangements caused by the alkyl chain itself.^125,126^ In order for an algorithm to understand and model such complex behaviors, an adequate number or such examples in the training set is needed.

Although the appropriate size of the training dataset is very much model- and problem-specific, there are some general rules that are good for every scientist to know. Generally, a ‘too small’ training set will result in poor data prediction. A model with too many correlation parameters will overfit a small training set. On the other hand, a model with far fewer correlation parameters than needed to describe the property, is likely to underfit the training set. In both cases, the result will be predictions with high degrees of uncertainty, whose performance will significantly depend on the similarity of the test to the training set.^127^

### Diversity

The case of imbalanced datasets is a very common problem in data science.^128^ In IL research imbalanced datasets can occur when the experimental data for one family of ILs (which is usually the alkylimidazolium ILs) significantly outnumber the other families. Most standard ML algorithms assume as default a properly balanced dataset and therefore it is possible that the model fits better the majority samples, while the minority cases are prone to major classification or prediction errors.^129^

The concept of balanced datasets is the direct response to ‘the bigger the dataset the better the algorithm will perform’. It is very important to keep a balance between creating a large and a diverse training set. Until recently, the IL community has mostly focused on alkylimidazolium salts, while other IL families came to the forefront only later. As a result, it is very common that available physical data on alkylimidazolium ILs dominate over the others. However, creating a dataset that has over 60% data on these ILs alone, leads naturally to the algorithm overfitting on these data, giving more accurate results on imidazolium salts, but producing higher uncertainty for the other ILs. Relevant examples of this under-representation can be found in the works of Baghban *et. al*.^101^ (65% of the dataset on imidazolium ILs and the rest on different families) and Song *et. al*.^107^ (only 1 sulfonium IL from the 124 ILs of the dataset). Hence, it is always important for the reader take note of the authors description of the dataset, so that they are aware of the limitations imposed by its composition and to not over-interpret the results.

Under-representation can also exist even within an IL family. The distribution of atoms in the ILs significantly affects the chemical interactions of their ions, resulting in isomers with different physical properties. Characteristic examples are the 1-ethyl-2,3-dimethylimidazolium and 1-propyl-3-methylimidazolium bistrifluoromethylimide ILs ([C_2_C_1_C_1_im][NTf_2_] and [C_3_C_1_im][NTf_2_], respectively), which although they are structural isomers, have very different melting points, with [C_2_C_1_C_1_im][NTf_2_] being solid at room temperature and [C_3_C_1_im][NTf_2_] having a melting point below −40 °C. In order for the algorithm to be able to correlate the properties to the given structures and make accurate predictions in such cases, all types of isomers should be equally (or at least comparably) represented in the datasets.

In order to make our case about under-representation of ILs clearer, we estimated the whole chemical space of isomers that encloses a specific dataset. We implemented Pólya's method to enumerate the number of isomers for acyclic alkyl chains as a function of the number of carbon atoms, which was taken from Fujita's work.^130^ This method does not take into account stereoisomerism (enantiomers and diastereomers), and therefore, the number obtained thereof represents only the lower limit of the total numbers of possible isomers. However, highly strained branched alkyl substituents which might not be thermodynamically stable, such as those analogous to *tert*-butyl, were not excluded from the count, but they represent only a marginal fraction of the total.^131^ Details about the enumeration method are further discussed in the ESI.†

**Fig. 7:**
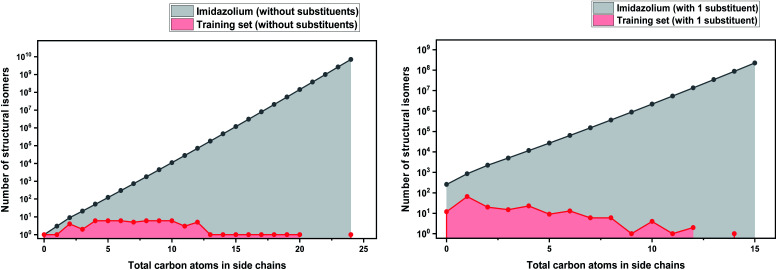
(a) Logarithmic plot of the isomer count for imidazolium cations in the work of Paduszyński;^85^ (b) taking into account the 86 different functionalized substituents that are shown in the paper.

**Fig. 8:**
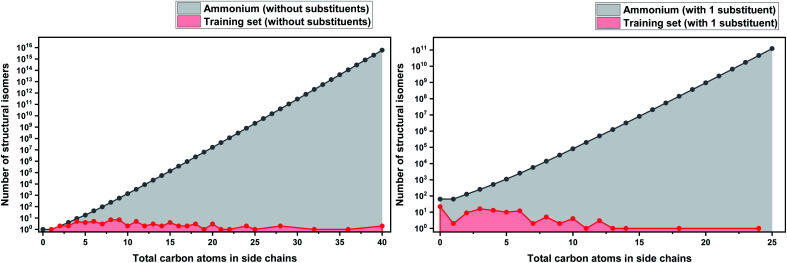
(a) Logarithmic plot of the isomer count for ammonium ILs in the work of Paduszyński;^85^ (b) taking into account the 64 different functionalized substituents that are shown in the paper.

As a basis for this analysis we used the work of Paduszyński,^97^ as it is one of the largest and most diverse datasets of all the published works. Fig. 7 and 8 show the cases of two of the most widely studied families of ILs, imidazolium and ammonium-based cations. The profile is very similar for all the presented cases, for smaller numbers of side-chain carbons (<4 carbons) the training set occupies a satisfactory percentage of the chemical space (in some cases up to 70%), while for larger numbers of carbons (>10 carbons) typically there are only a couple of studied IL. This behaviour is expected, as longer-chain ILs are usually more difficult to synthesise, so the available physical data on those are very limited.

To understand the impact of this, the algorithm will try to predict a chemical space of 10^8^ ILs, based only on 1 or 2 representative examples. As a result, the model will probably try to extrapolate the behaviour of these isomers from the behaviour of the better-represented small carbon number space. Here we face a very interesting question, will a change in the distribution of carbons on an alkyl chain affect the properties the same way for an IL with 10 carbons as for an IL with 4? Will changing the distribution of carbons on an alkyl chain affect the properties of a low- and a high-molecular weight IL in an analogous way? To our knowledge there is no available published work responding to these questions, therefore it is unknown whether making the assumption that they will can be safely used for the extrapolation of the behaviour of ILs. As will be discussed further below, extrapolation is not a wise choice in ML models, especially when based on such uncertainties. It is also important to point out that in our calculations we only explored the chemical space created by the structural isomers of the cations. Since the properties of ILs come as a result of cation–anion combinations, by introducing different anions the chemical spaces are automatically increased by many orders of magnitude. The number of structural isomers (excluding enantiomers and diastereomers) for the imidazolium cation with a total of 55 carbon atoms with acyclic alkyl chains substituents only is already in the order of magnitude of the Avogadro number (∼10^23^), this implies that the chemical space of ILs is astronomically large.

### Consistency

IL prediction methods rely heavily on experimental data. It is extremely time consuming for scientists to synthesize an IL from scratch and measure its properties of interest, in order to create their own datasets. Therefore, we start by looking in the literature in order to collect as much as possible of the required data. However, comparing measurements from different works, requires that the researchers have deep understanding of the methods used and whether they are indeed comparable.

A characteristic example of such a case is rheology. Viscosity is a widely studied property, especially for ILs (see Table 1). ILs that are too viscous are generally not industrially preferable, therefore accurate prediction of the ILs' viscosities has great economic value, and can reduce the need to synthesise many ILs in order to find one with a suitable viscosity. There are many different techniques for viscosity measurements (*e.g.* dropping ball, flow cups, capillary and vibrational viscometers), but are all relative measuring systems. The obtained results are highly dependent on the instrument's architecture and they can't be simply compared to each other.^132,133^ Absolute measuring systems, which don't depend on the size and shape of the device, can provide the researchers with absolute viscosity values, but they are based on specific standards, such as DIN 53019 or ISO 3219.^134^ It is very common among the studies that we have cited in this work that they create their datasets from a large variety of published works, without taking into account the technique or conditions applied to each study. As a result, the consistency of the datasets is compromised.

Many of the studied properties in ILs, such as gas solubilities, density and viscosity are dependent on the experimental conditions, such as temperature and pressure. In order to achieve high prediction accuracy, it is very important to maintain dataset consistency throughout the dataset concerning any such parameters. Let's take the hypothetical scenario where a training set is created from 2 papers measuring the solubility of a gas in different families of ILs. If the two subsets have been measured over different pressure ranges, then the ML algorithm will overfit the non-overlapping range for only one of the families. Therefore, the mid-range pressure predictions will be based upon data from both families of ILs and be more generally applicable, but the start- or endpoints will be based upon data from just one of the families of ILs and will likely not give accurate results beyond this family. Therefore, it is important to filter the dataset in order to include the same range of parameters from the experimental measurements. A characteristic example of this case could be observed in the work of Baghban *et. al.*,^40^ where the dataset includes viscosities of amino acid ILs only at 353 K, so the prediction of other temperatures will be based on approximations from the other IL families. Similarly, Fatehi *et. al*.,^99^ train their NN on 66 ILs, from which only 7 have experimental values above 373 K (all of these are methylimidazolium ILs), so the predictions at these temperatures will be based strictly on those. Similar examples can be found in most of the works presented in Table 1.^84,113^

### Certainty of data quality

Last, but not least, a major issue in IL research, as well as in every ML application, is the quality of the available physical data.^135^ It is very common in the literature of ILs data for different values to be reported for the same property for a particular IL. A very characteristic example of that is the melting point of a very commonly studied IL, [C_2_C_1_im][BF_4_], for which the available data vary from 5.8 to 16 °C.^136^ There are also many studies of reaction kinetics, which is another domain where ML methods could be useful,^137^ that show that common impurities, such as moisture or unreacted starting materials can significantly affect the results.^138^

In order for results to be reproducible, the ILs have to be either ultrapure (<0.1% of impurity levels)^139^ or the level of purity has to be clearly stated in each work. This has become more common in works published in the last few years, but there are minimal purity data from earlier IL research, which unfortunately makes their use very limited without re-testing the results. For example, it is quite common, especially when synthesized at high temperatures, for ILs to have a characteristic red-brown colour. A lot of effort is taken, *e.g.* by multiple recrystallizations or treatment with activated charcoal, to remove the colour from the salt. However, the origin of these colours is still a mystery, since these ILs do not show any distinct impurity peaks in IR or NMR and, often, they may not affect the properties.^138^ Receiving a colourless IL is very often used as an indication of purity. However, as many of the property-affecting factors, such as metal ions, halides or water, don't add any color to the IL, they need to be quantified separately.^140^

This causes a major issue when selecting ILs for the training datasets. One way of dealing with the issue would be to consider each impurity as an independent factor affecting the physical properties and try to integrate it in the prediction model. However, this would make the algorithm very complicated and, to our knowledge, this hasn't been implemented by any researcher so far (probably due to the lack of enough data on impurities). Most researchers manually handle their datasets, by excluding data points that seem as outliers or by just trusting that the ILs in the published works are pure enough. Manual handling of data is problematic by default, because it is not easy to handle thousands of data points and sometimes, especially when predicting gas solubilities in ILs, the outliers are not as apparent as in the case of viscosity or density.

**Fig. 9:**
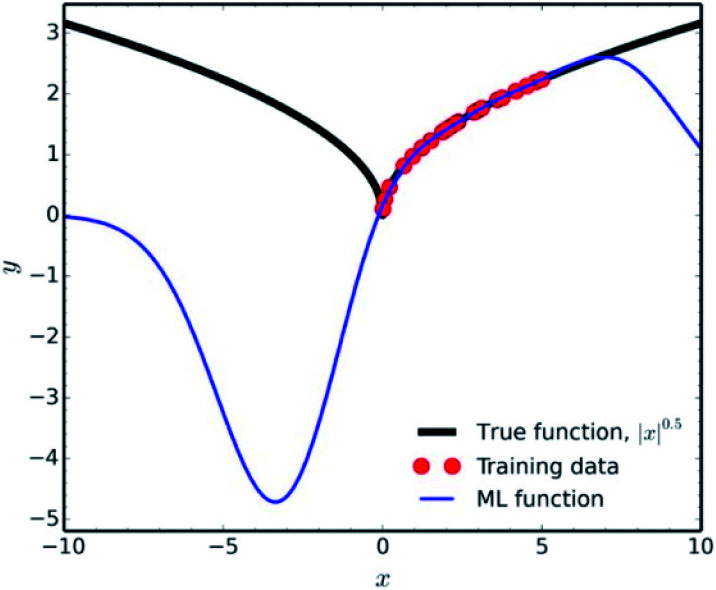
Interpolation *vs.* extrapolation with ML of the function |*x*|^0.5^ (black line). ML predictions (blue line) were obtained with kernel ridge regression trained on 25 randomly drawn points (red dots) from *x* ∈ [0; 5]. Reprinted with permission from Pavlo Dral.^141^ Copyright 2020 American Chemical Society.

Another factor which falls under the data quality category, is how representative is the training set of the studied chemical space. It is fundamental in data science to use the ML results only for interpolation of experimental values. Extrapolation is not a good practice, since many common ML methodologies function as ‘black-boxes’, the researcher can never be certain of the true equation hidden behind a NN. A very characteristic example of the poor extrapolation potential of ML is presented by Pavlo Dral for the simple function of |*x*|^0.5^ (Fig. 9).^141^

For ILs properties prediction analogous cases would be predicting properties for shorter/longer alkyl chains, or lower/higher temperatures, than the training set's threshold, introducing new functional groups *etc* (see Diversity subsection). Achieving high accuracy in those types of predictions would be a matter of luck, rather than an efficient algorithm.

Showing the extrapolation incapability of ML in a simple mathematical function as the one described above, should raise major concerns for extrapolation in complicated chemical spaces. Collecting appropriate training data for high-dimensional spaces, such as chemical space, is a major problem in data science because of the so-called ‘Curse of Dimensionality’.^142^ There are various methods that are being used in order to minimise the amount of training data needed and reduce the data extrapolation as much as possible (such as farthest point sampling and structure-based sampling), but the readers should refer to more relevant literature for information on those.^143,144^

The difficulty in extracting consistent, high quality data from the literature leads to the possibility of collecting bespoke data sets as inputs for ML approaches. Recent years have seen incredible advances in high throughput experimental techniques.^145–147^ Attempts have been made to apply high throughput techniques to the measurement of physical data for ILs^148–150^ and to couple this with ML.^151^ However, the range of ionic liquids to which this has been applied has been restricted by the multistep synthesis and complex purification that many ILs require. Hence, these attempts have been restricted to those ILs that are synthetically more accessible, such as protic ILs.^152,153^ As has been described above, one cannot simply extrapolate these results to other families of ILs. Another very useful alternative is the design and use of automated robotic platforms, which could synthesise and/or test the physical properties of the studied systems.^154^ These platforms, although they are capable of collecting huge amounts of data in short times, in the case of ILs would still be delayed by synthesis and purification procedures.

Interestingly, there is a well-known methodology, which could support the more accurate implementation of ML algorithms, and this is Design of Experiments (DoE). There are several studies, unrelated to chemistry research, which use DoE frameworks to fine tune the selection of initial hyperparameters and reduce in general the complexity of ML tuning.^155,156^ On the other hand, ML could substantially help the aim of DoE by detecting non-obvious factor effects and interactions (falsely considering interrelated factors as independent is a common problem in DoE approaches).^157^ ML algorithms could completely replace the DoE approaches, as theoretically they are able to create correlations by taking into account all the possible factors influencing a process. However, in reality we are significantly restricted by the lack of enough computational power (and sometimes data) to create and run such complex models. Therefore, the combination of the two methods is indeed relevant and will keep being useful for the foreseeable future. Over the last few years, combinations of ML and DoE have been used to optimise materials design^158–160^ or various synthetic procedures,^161,162^ however to our knowledge this hasn't yet been expanded to the IL area.

## Machine learning for molecular dynamics simulations

**Fig. 10:**
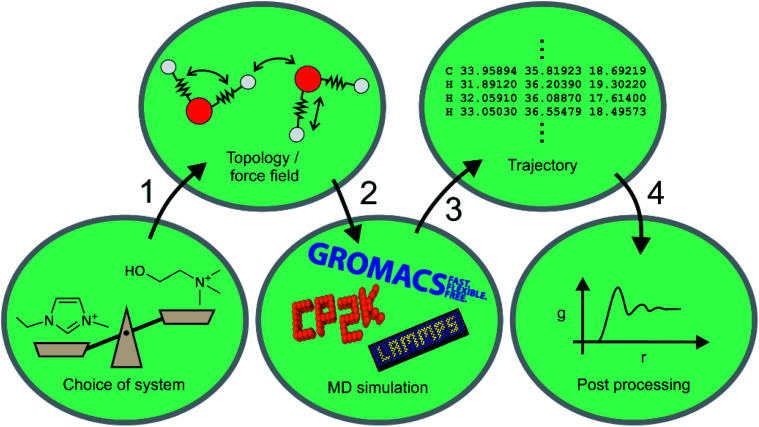
Typical steps of an MD simulation.

Machine learning still has to gain traction in the ionic liquid community. In this section, we will compare machine learning to a well-established theoretical method, that of molecular dynamics (MD) simulation. Molecular dynamics uses numerical integration of Newton's equations of motion to predict how the positions of atoms (or groups of atoms) evolve over time. Statistical thermodynamics is then used to derive macroscopic properties, both structural and dynamic. An MD simulation thus typically consists of the steps shown in Fig. 10, and ML can be used to enhance virtually every aspect of MD. Naturally, ‘Machine Learning’ is a much more general term, and encompasses methods that can be seen as a sophisticated tool for fitting and statistical analysis. We will give a brief overview here of the use of molecular dynamics in ionic liquids, how it differs from machine learning methods, and how the two approaches can be used synergistically. The ML examples we present are largely from outside the field of ionic liquids, but the general concepts can and undoubtedly will be used for ionic liquids as well. A good overview of the approaches presented in this section can be found in ref. 141, ^167–169^.

Over the past two decades, MD simulations have substantially advanced the understanding of ionic liquids by modelling the structure and dynamics of the liquid phase.^170–172^ Many ionic liquids, in particular those with long alkyl or perfluoroalkyl side chains, show pronounced nanosegregation into polar, non-polar, and in some cases fluorous domains.^172^ MD simulations provided invaluable insight into how and when these domains form.^173–180^ Even in cases where the liquid structure can be probed experimentally with scattering experiments, MD simulations are required to trace back the observed features to structural motifs on the molecular scale.^181,182^ One of the crucial advantages here is that MD simulations based on classical force fields allow for targeted modifications which are not possible experimentally. For example, several groups used MD simulations with artificial, deliberate changes in the dihedral parameters to increase the barriers for rotation around specific bonds, thus separating out the effects of conformational flexibility.^183–186^

Despite the astounding successes of classical MD simulations, one of the central problems remains the choice of a force field, *i.e.* the first step in Fig. 10. MD simulations rely on the availability of accurate forces and energies as a function of atomic positions. For ionic liquids in particular, polarizability is more and more recognised as an essential element for the accurate prediction of structure and dynamics.^178,187–192^ It is to some degree possible to mimic the effects of electronic polarizability with scaled charges, however this comes at the expense of lost accuracy.^193,194^ Even in cases where explicit treatment of polarizability is not necessary, choosing a reasonable set of atomic charges along with well-balanced bonded parameters is a nontrivial task.^195–199^ The vast number of possible ionic liquids is yet another serious challenge for force field development, and transferable force fields are required to not be limited to one particular system.^195,200–207^

Molecular dynamics simulations can be used to predict a wide range of properties of ionic liquids from thermal transitions to transport, structural, or spectroscopic properties.^74,199,208–216^ The prediction of properties with MD simulations has two facets. First, the predicted property can be compared with known experimental values to validate the method or force field, similar to the test sets for ML algorithms.^217,218^ Properties such as density, self-diffusion coefficients or surface tension are commonly used for this purpose.^210,211,215,219,220^ Good agreement between experiment and MD simulation suggests that the relevant physics are reasonably reflected by the model, which is then used to either gain mechanistic understanding or to predict a different property. The second facet is thus the use of MD simulations to predict hitherto unknown properties. Similar problems to ML methods arise in the sense that the more widely applicable models (*i.e.* generic classical force fields) perform poorly for quantitative predictions, whereas interpolation of properties of similar compounds can be done much more reliably. An exception are *ab initio* MD simulations, which do not rely on a force field and can be used to predict ionic liquid properties, if sufficient computational resources are available.^74,208,209,221^

**Fig. 11:**
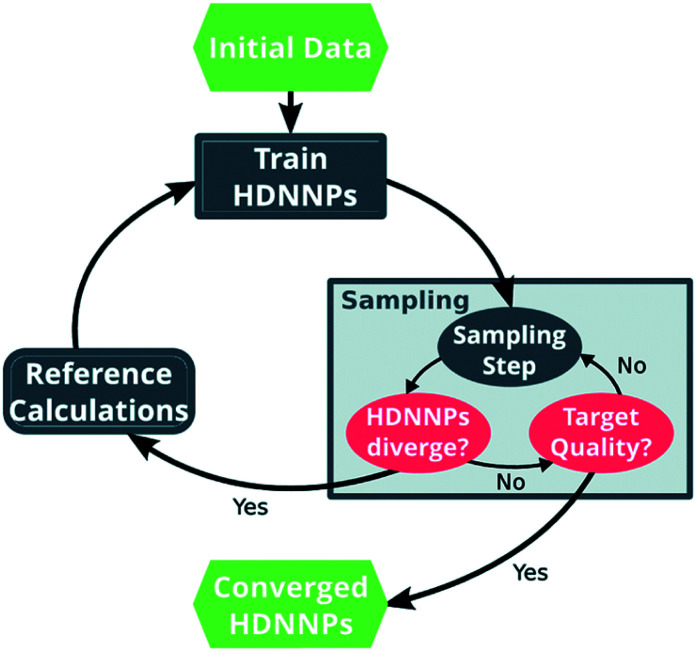
Scheme of the general approach to automatically construct a force field using ML, in this case HDNNP. The ML algorithm is trained using the output (forces, energies) of a more expensive higher level method. The simulation is evolved using the MLIP, and re-trained every few steps to avoid extrapolation. Once converged, the computationally inexpensive MLIP can be used for production purposes. Reprinted by Gastegger *et al.*^233^ – published by The Royal Society of Chemistry.

One way in which MD simulations and machine learning can be used synergistically is to automate the construction of force fields, an otherwise complex and laborious task. Broadly speaking, machine learning as an advanced ‘fitting tool’ can be used to obtain a force field by fitting forces and/or energies.^167,222–225^ Thus, machine learning interatomic potentials (MLIP) are usually trained on high-level *ab initio* methods to yield accurate energies (and forces) as a function of atomic coordinates.^226–230^ An example are High Dimensional Neural Network Potentials (HDNNP), which aim to fully replace the *ab initio* method once trained.^222,231–233^ The MLIP can be re-trained ‘on the fly’ every few steps using a high level *ab initio* method.^233–235^ This implementation avoids the issues associated with extrapolation (as described in the previous section), a good illustrative example is given by Botu and Ramprasad,^235^ as well as in Fig. 11. However for all MLIP, some effort has to be made to incorporate physical constraints such as conserved quantities or invariance with respect to rotation and exchange of identical particles.^167,223,236^

Purely *ab initio* molecular dynamics – as opposed to those based on classical force fields – become more and more feasible for ionic liquids, but remain computationally expensive.^205,208,237^ Machine learning can be of use to enhance and accelerate the quantum chemical method itself, rather than providing a complete substitute such as in MLIP.^168,238^ For example, a Δ-learning scheme can be used which learns only the difference between a cheap low level method (semi-empirical, classical, *etc.*) and an accurate high level method (DFT, post-HF *etc*.).^168,239–242^

Just as important as the simulation itself is the final step shown in Fig. 10, *i.e.* the post processing of the trajectory. Analysis tools such as TRAVIS^243,244^ are invaluable to extract structural and dynamic information from a trajectory which by itself does not provide information to a human reader. Purposeful post processing and visualisation is crucial to understand the behaviour of bulk ionic liquids by means of MD simulation.^245–247^ MD simulations can thus serve as a bridge between molecular features and bulk properties.^248,249^

The high dimensionality of an atomistic trajectory can in some cases be reduced to just a few dimensions which can be understood by a human. Such low dimensional collective variables have already been used to describe nucleation and solute conformations in ionic liquids.^250–252^ ML can be employed to find collective variables to describe complex transitions, which can then be used to bias and analyse the system.^169^

Furthermore, there are several studies where machine learning has been used to extract information from or in combination with an MD simulation. In a recent publication, Jung and Yethiraj used a deep neural network DNN to predict the phase diagrams of mixtures of ionic liquids with poly(ethylene oxide).^253^ An example outside the ionic liquid community is the decomposition of 1,2-dioxetane, which has been investigated using *ab initio* MD simulation.^254,255^ Machine learning models were then used to identify the required conditions for different decomposition pathways and lifetimes.^254,255^ This example shows that machine learning can indeed provide conceptual insights.

To conclude this section, we would like to consider the bigger picture, *i.e.* the purpose of the process shown in Fig. 10. Many MD simulations in the ionic liquid community are used to understand a well characterised system, rather than as an actual prediction tool for the unknown. Machine learning, on the other hand, is often used as an interpolation or ‘fitting’ tool trained on an experimental database. However, ML and MD can also be combined to take advantage of each. For example, MD simulations are well suited to study electrostatic screening in ionic liquids.^256,257^ Although not specific to ionic liquids, Kadupitiya *et al.* developed a ML model to predict the ion density profile of a confined electrolyte.^258–260^ The ML model was trained on MD simulations and takes simple parameters as input, such as the concentration of a salt, the confinement length, or the ion diameters.^258^

Machine learning can be used to enhance molecular dynamics simulations and *vice versa*. The examples outlined above show the great benefits of such a synergistic combination, exploiting the strengths of each method and avoiding their weaknesses. It is without doubt that the exciting advances made by machine learning will be used increasingly by the ionic liquid community, once knowledge spreads and the required algorithms become implemented in common software packages. Machine learning promises faster and more accurate simulations as well as new tools for the interpretation of results, and the future will show to what degree these promises translate to practise.

## Future aspects

Research to date on the applications of ML algorithms to ionic liquids has proven that these are competitive with other computational algorithms in terms of classification and can provide excellent prediction capacity (within the constraints described above). Indeed, the majority of studies (see Table 1) had this as their primary objective, or in some cases to compare the effectiveness of different ML approaches to provide such predictions. However, there is much more they can offer. ML models generally show a trade-off between transparency of their decision-making process and the accuracy of prediction. For example, DTs offer incredible possibilities for the user in terms of understanding and post-processing the decision making process, however they are not able to generate such complex correlations as DNNs – which in their majority still have to be considered as ‘black boxes’ and be trusted without investigating how they reached a result.^261,262^

Understanding the intermediate steps of the decision-making process could prove extremely beneficial for the IL research field. Working in the basis of physical sciences research, researchers are trying to interpret the natural phenomena and model them mathematically in order to predict the behaviour of the studied, as well as unknown systems. ML can help with that, because it offers the advantage that it doesn't need to understand chemistry in order to detect correlations. Given a dataset of independent measurements, we can train an algorithm that will eventually manage to identify the relevant features that significantly contribute to the studied property.

Explainable AI (XAI) refers to the process of creating AI models which use interpretable parameters as part of their decision-making process.^263^ The significance of this is enormous, starting with data protection and copyrights. As per 2018, according to General Data Protection Regulation (GDPR) citizens of EU are granted the “right to explanation” if they are affected by a decision-making algorithm.^264^ Obviously, this right cannot be claimed when the complexity of an AI algorithm obscures the rationale behind the recommended decision.

XAI practises can have a significant impact on chemical research, as they can help researchers to improve their understanding and knowledge on the investigated properties or processes.^265^ In IL research there have been some initial attempts to explain the effect of specific parameters for simpler (first order) linear regression algorithms.^107^ Greaves *et al.* used two different ML algorithms, a NN and a multiple linear regression algorithm to predict the reaction rate of a bimolecular nucleophilic substitution in different ILs. In their work they showed that, although NN gives the best statistical fitting, it doesn't give the possibility of judging which descriptors are significant. On the other hand, the linear regression algorithm, which also provides adequate results, clearly shows which descriptors mostly affect the model.^56^ According to their study, the reaction rate is mostly affected by three cation descriptors, namely the number of secondary sp^3^ hybridised carbons, the number of rotatable bonds and molar refractivity.

While using first-order models allows easier understanding of the significant contributions to any property, due to the simplicity of their nature; the same simplicity means that this comes at the cost of lower accuracy in predicting complex behaviours, such as viscosity. Low *et. al*. very accurately state in their work that many semi-empirical predictions could likely be refined by using a higher level of theory during initial parameter selection, instead of using the arbitrarily-engineered features that are popular in many models.^92^ In practise this would mean choosing IL descriptors that are based on distinctive properties (such as HOMO–LUMO gap, or σ-profiles) instead of an artificial representation that has no meaning in physical space (such as SMILES descriptors).

On the other hand, explaining the parameters of non-linear correlations, such as those easily detected by NNs is far more difficult. Paduszyński has effectively modelled the viscosity dependency of a very large dataset of ILs, concluding that the interpretation of the resulting parameters is not practically feasible. He even argues that these models might not be useful for evolving our fundamental knowledge of viscous behaviour of ILs.^97^ Probably the most significant part of creating explainable models is the representation of the initial data, arbitrary representations almost certainly will result in non-transparent and non-interpretable models. There are various systems trying to achieve *post-hoc* or *ante-hoc* explainability of decision making, in order to increase the scientists' trust towards AI.^266^ Pflüger and Glorius mention that in order for us to understand what machines learn “XAI must find its way into chemistry”, which requires adequate understanding of both the algorithms and the chemistry.^267^ Indeed, extremely complex algorithms that are only understood by a specialised computer scientist and input data that are only understood by a theoretical chemist are the bottleneck for the progress of this field. Such systems have started being implemented in several chemistry-related studies,^265,268,269^ but they are still not popular in IL research. Recent work by Ding *et. al.* implemented the shapely additive explanation (SHAP) method, in order to interpret the models and quantify how each parameter affects predictions.^270^ Their work is a valuable step towards XAI in ILs.

**Fig. 12:**
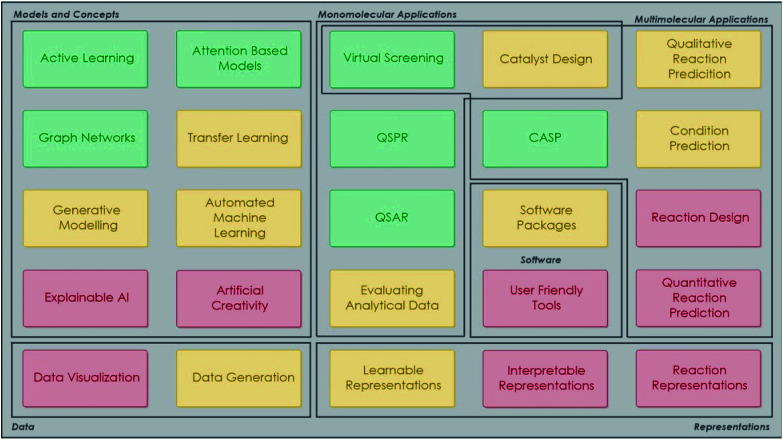
Subtopics of ML applications for chemistry research, categorised by the number of published works. Red: highly underexplored; yellow: some attempts demonstrated; green: fields of major attention. Reprinted by Pflüger and Glorius^267^ – Published by John Wiley & Sons.

It is true that there is an infinite space of unexplored possibilities to use AI not just for predicting, but also for enhancing our understanding of ‘hidden’ factors affecting physical and chemical processes, which is yet not reachable (Fig. 12). However, in the near future, these problems will be overcome. New generations of scientists will be far more familiar with those methods and will be more multidisciplinary trained and able to analyse and understand the results. The preliminary work that is currently conducted will create a solid basis in order for deeper exploration and understanding of the underlying knowledge.

## Concluding remarks

Machine learning has recently become a widely studied field used for understanding material phenomena.^271^ Its superior classification and prediction capabilities make ML algorithms an extremely useful tool for computational scientists of all disciplines, as they are able to analyse enormous datasets in short times.^272^ Regarding chemical research, ML-based methods have heretofore been mainly used for property prediction for polymers^273^ and pharmaceuticals,^274^ systems of high economic significance which are also thoroughly studied experimentally.

Over the past few years, ML-based research has expanded to complex ionic systems and, eventually, to ILs. The majority of published works in this field explore the use of ML techniques either for the prediction of their physical properties, or for solubility of gases in ILs, with the purpose of the study being to demonstrate that ML can be a useful tool. Others have compared how different ML algorithms have performed for particular predations. In this review we collected and discussed the available literature on the use of ML in the ILs' field, and have noted the impacts of common problems with the literature of ILs physical properties, such as the diversity of ILs that have been studied and the quality of the data. These compromise the quality of the datasets available and, as a result, limit the scope and quality of the possible predictions.

At this point it is important to note that we intentionally did not attempt quantitative comparison of the accuracy across different models. To be able to do this, it is very important to compare the performance of multiple different ML algorithms consistently. This is not always easily performed by the data supplied in a scientific paper, as there are numerous different accuracy indicators and their use is not consistent across different works, with each researcher having different definition of a successful model. Moreover, in order to conclude which model is superior, we would need to train them on the same dataset and test them on the same test set. Datasets are often biased, which means that the nature of the dataset makes the model perform better or worse in specific cases (*e.g.* perform better for imidazolium than phosphonium ILs).^275^ Also, the test sets in most cases are derived by excluding some ILs from the training set, so the tested examples are not completely independent from the training set, as they come from the same set of experimental measurements. In order to compare and judge the performance of the algorithms, one would have to encumber them with the same bias (same training set) and the test their performance on a truly independent, randomly selected test set. Creation of standardised, unbiased and truly independent datasets for training and testing algorithms is something that has been widely studied in many other fields of computer science and ML research, but not yet for ILs.^276^ This is primarily, as discussed in above, due to the lack of many trustworthy physical data for ILs and also, since the synthesis and study of ILs is usually hard and time-consuming, such work would require incredible effort and collaboration of many researchers.

Furthermore, we tackled another interesting point of ML application, ML-enhanced MD simulations. The majority of works on this area use ML methods to automate the production of input parameters for MD simulations (*i.e.* force fields, quantum chemical calculations) or for post-processing of the resulting trajectories, taking advantage of the classification and statistical analysis capabilities of such algorithms. This results in faster setup and analysis of MD simulations, but doesn't fully utilise the ML's prediction capacity. Therefore, it is apparent that ML methods show great potential, not as antagonists, but rather as enhancers of MD simulations. There also seem to be some initial attempts to combine ML and MD methods to predict behaviours of non-experimentally characterised systems, which however have not expanded to IL research. This could eventually lead to exceptional results, however it is still early days and such research requires collaboration of interdisciplinary teams with high expertise in both computer science and computational chemistry.

Finally, we would like to conclude this work with a look into the future. All the cases described above are about the simplest case of having neat ILs. However, there is the growing interest in using mixtures of ILs with molecular solvents or other ILs in order to overcome common problems (such as high viscosity).^277^ However, these new solvent systems are extremely complicated and require a thorough characterisation on their own. Optimising such systems creates a complex chemical space, whose exploration dramatically increases the number of experimental measurements, as changing the composition of the mixture dramatically alters its properties. Therefore, there is an urgent need to minimise the number of samples that are needed in order to have an accurate representation of the space (DoE and high throughput screening).^278^ There are only limited published works on ML-assisted screening of such complex mixtures,^151,279–281^ but this is certainly one of the areas where ML models can flourish.^282^ Similarly to the case of MD simulations, combining different methods can certainly enhance their capabilities, but requires a great amount of expertise and interdisciplinarity. Someone could say that we are still in the prehistoric period of ML-aided research, although much effort is given in order to include such models in commercial software packages. One thing is certain, once ML models become broadly available to users, they will completely change data analysis and experimental design. Automated robots that perform complex tasks, while getting feedback from ML models in order to improve their output have already been created and show extraordinary results.^283^

## Author contributions

Conceptualization S. K., T. W.; supervision T. W.; writing – original draft preparation S. K., F. P., F. M.; writing – review & editing, S. K., F. P., F. M., T. W.

## Conflicts of interest

There are no conflicts to declare.

## Supplementary Material

SC-012-D1SC01000J-s001
