# Supplementary material for: A review on machine learning algorithms for the ionic liquid chemical space
Source: Chem Sci. 2021 May 6;12(20):6820–43. doi: 10.1039/d1sc01000j (PMC8153233; doi:10.1039/d1sc01000j)
Supplement: SC-012-D1SC01000J-s001 [file SC-012-D1SC01000J-s001.pdf]

Electronic Supplementary Material (ESI) for Chemical Science.
This journal is © The Royal Society of Chemistry 2021

Electronic Supplementary Information

A Review on Machine Learning Algorithms for the Ionic Liquid Chemical Space

Spyridon Koutsoukos [a], Frederik Philippi [a], Francisco Malaret [b] and Tom Welton [*a]

[a.] *Department of Chemistry, Molecular Sciences Research Hub, Imperial College London, White City Campus, London W12 0BZ, UK.*
[b.] *Department of Chemical Engineering, Imperial College London, South Kensington Campus, London, SW7 2AZ, UK*
* E-mail: t.welton@imperial.ac.uk

The isomer enumeration for saturated acyclic alkyl chains substituents has been performed using Pólya's method for monosubstituted alkanes, in which the substitution group is the linkage to the cation core. The computational implementation of Pólya's method to estimate the number of isomers for alkyl chains as a function of the number of carbon atoms was taken from Fujita's work [1] (original reference in German, not consulted [2]). This method enumerates only structural isomers, ignoring stereoisomerism (enantiomers and diastereomers). Therefore, the number obtained thereof represents only the lower limit of the total numbers of possible isomers. An example of the isomers enumerated for 6 carbon atoms is depicted in Figure S1.

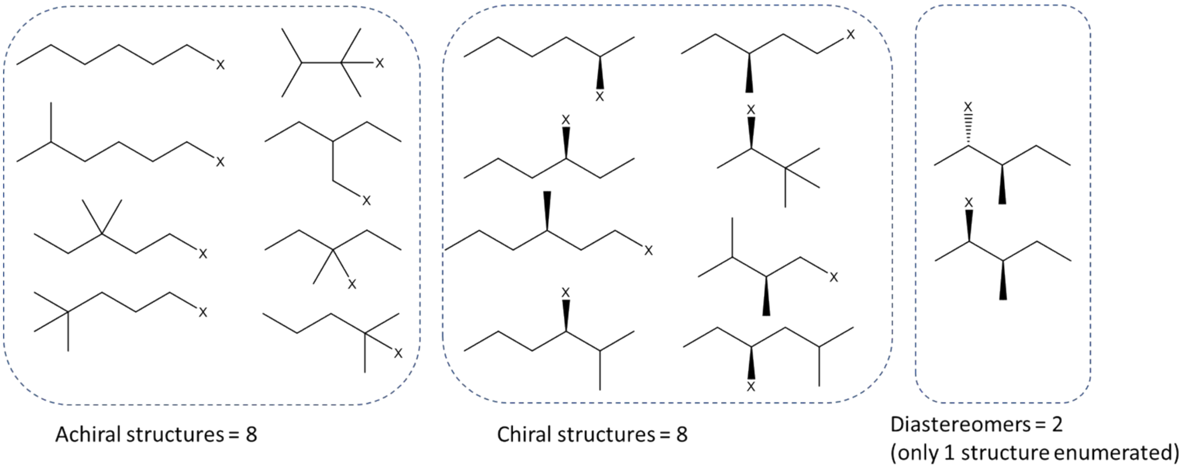

Figure S1. Structural isomers for acyclic alkyl chains containing 6 carbon atoms. X represents the attachment point to the cation core. Pólya's method does not discriminate between enantiomers and diastereomers, therefore, the total number of structural isomers for C6 is 17.

If stereoisomerism is taken into account, the total number of possible structures will significantly increase with an increasing number of carbon atoms. However, highly strained branched alkyl substituents which might not be thermodynamically stable, such as those analogous to tert-butyl, were not excluded from the count, but they represent only a marginal fraction of the total. [3]

Enumeration of the number of isomers with alkyl substituents was performed by replacing the Rx substituents in the structures shown in Table S1 with different acyclic alkyl isomers calculated with Pólya's method.[1,2] The total number of structural isomers was then counted, with a python implementation, as a function of the total number of carbon atoms in all side chains (Rx). The first isomer corresponds to the protic version of the cations, in which all the Rx are hydrogen atoms. An example of the enumeration method for the imidazolium cation up to a total number of carbon atoms of 2 is shown in Table S2.

Table S1. Chemical structures for the cations used in the datasets presented in Table 1 and Table 2 in the main text.

Cation Names	Structure
Im: Imidazolium	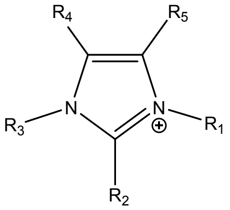
Py: Pyridinium	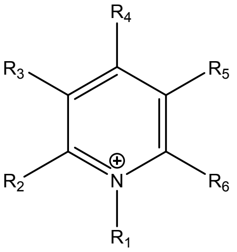
Quin: Quinolinium	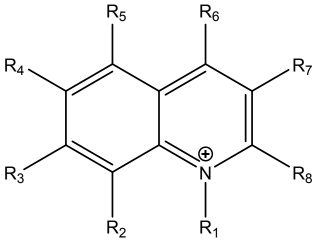
Pyr: Pyrrolidinium	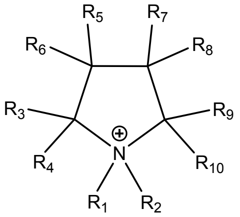
Pyrr: Pyrroline	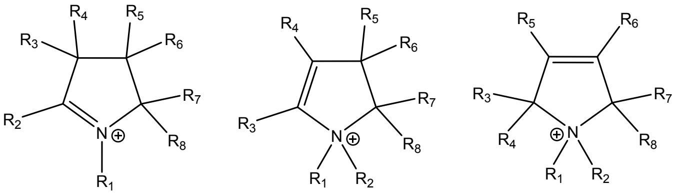
S: Sulfonium	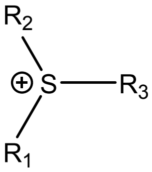
Ox: Oxazilidinium	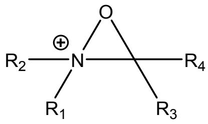
Pip: Piperidinium	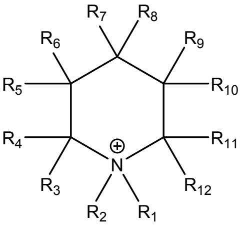

Cation Names	Structure
Mo: Morpholinium	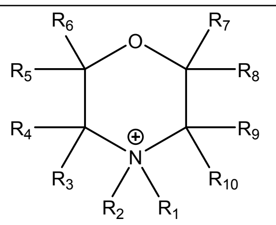
Azp: Azepanium	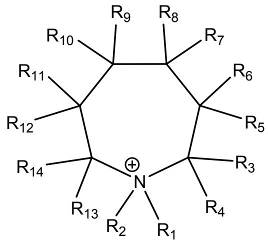
N: Ammonium	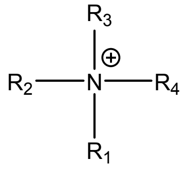
P: Phosphonium	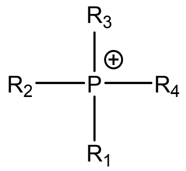
Cprop: Cyclopropanium	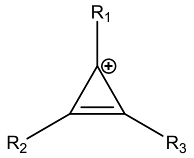
Guan: Guanidinium	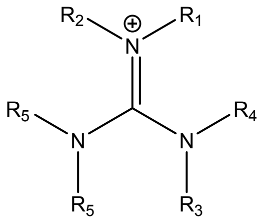
Trz: Triazolium	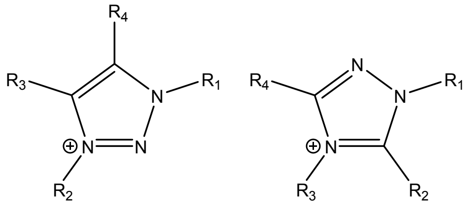
Bic: Bicyclic	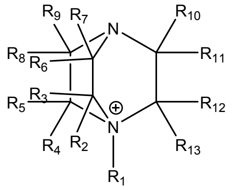
Pz: Pyrazolium	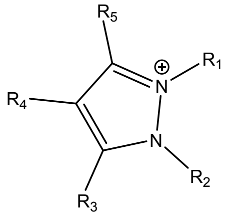

Cation Names	Structure
Thur: Thiouronium	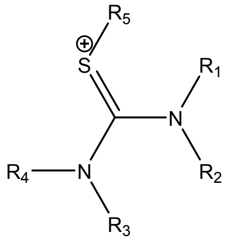
Cs: Cyclic sulfonium	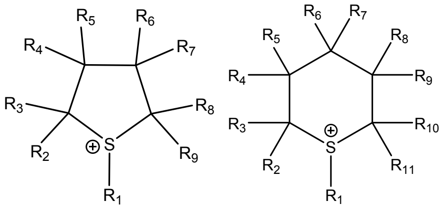
Thz: Thiazolium	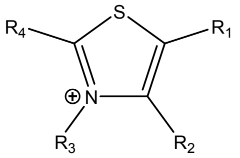
Amd: Amidium	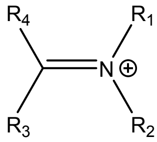
Pipz: Piperazinium	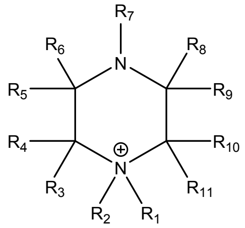
Tetraz: Tetrazolium	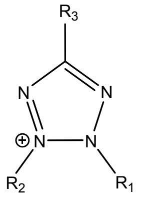
Ur: Uronium	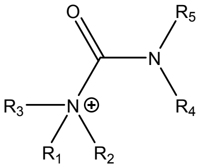
Guan: Guanidinium	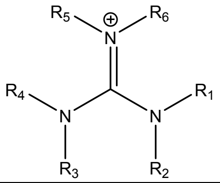

Table S2. Example of isomeric structures for the imidazole cation.

Total Number of Carbon atoms	Structures	Total Number of Structures
0	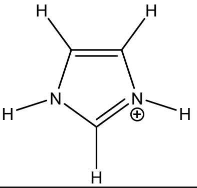	1
1	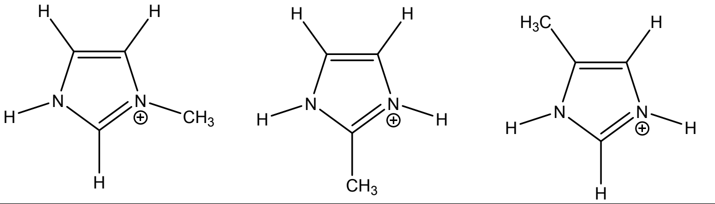	3
2	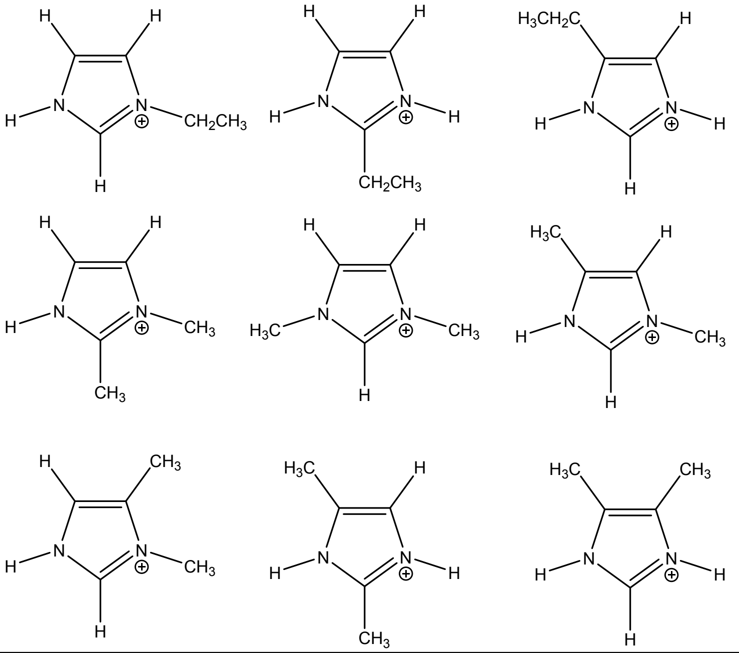	9

Functionalized substituents (those containing unsaturations, cycles, halogens, heteroatoms, or others) were considered as a whole, and their isomeric structures were not enumerated, only their relative positions within the cation (Figure S2). For structures containing both, complex groups and acyclic alkyl groups, the saturated alkyl group were permutated as the example shown in Table S2, up to the maximum number of carbon atoms of the alkyl chains. The number of isomers as a function of the total atoms of carbons in the substituent chains was then multiplied by the number of functionalised substituents within the data set. This estimates the minimum number of possible isomers, if isomeric structures for the substituents are considered, the chemical spaces will significantly increase. With more substituents, a similar approach was followed.

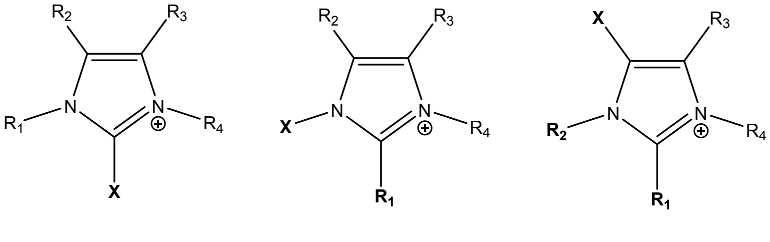

Figure S2. Example of imidazolium cations with 1 functionalised substituent (X). Rx represents acyclic alkyl chains.

Finally, an example of the minimum chemical space for the ionic liquids used by Paduszyński, one of the largest datasets for ILS, is shown in Table S3. [4] In this example, it can be seen that the number of ionic liquids used to train the algorithms cover only a very small fraction of the total chemical spaces.

Table S3. Comparison of the number of cations used by Paduszyński against the estimated minimum chemical space. [4]

Ion	Maximum total number of carbon atoms in side chains	Number of functionalized substituents	Number of different functionalized Substituents	Cations in dataset	Minimum number of total structures	Percentage
im	24	0	0	66	11339809016	5.8×10^{-7}
im	14	1	86	179	145036850	1.2×10^{-4}
pz	7	0	0	6	1244	0.48
pz	9	1	3	11	52731	0.021
trz123	9	1	3	6	52731	0.011
trz124	10	1	3	9	128862	7.0×10^{-3}
tetraz	1	1	1	1	3	33
bthz	0	1	1	1	6	17
dc-im*	4	1	45	45	10530	0.43
dc-n*	10	1	4	5	1768	0.28
n	24	1	64	105	4.75346×10^{12}	2.2×10^{-9}
p**	38	0	0	42	4.019×10^{14}	1.0×10^{-11}
P**	24	1	9	12	3530759355	3.4×10^{-7}

* dc: dication

** including protic tertiary phosphonium cations.[5]

References

1 S. FUJITA, *J. Comput. Chem. Japan*, 2007, **6**, 59–72.

2 G. Pólya, *Acta Math.*, 1937, **68**, 145–254.

3 R. S. Paton and J. M. Goodman, *J. Chem. Inf. Model.*, , DOI:10.1021/ci700246b.

4 K. Paduszyński, *Ind. Eng. Chem. Res.*, , DOI:10.1021/acs.iecr.9b00130.

5 U. A. Rana, R. Vijayaraghavan, M. Walther, J. Sun, A. A. J. Torriero, M. Forsyth and D. R. MacFarlane, *Chem. Commun.*, 2011, **47**, 11612.
